# Manganese exposure from spring and well waters in the Shenandoah Valley: interplay of aquifer lithology, soil composition, and redox conditions

**DOI:** 10.1007/s10653-024-01987-4

**Published:** 2024-05-02

**Authors:** Margaret A. G. Hinkle, Brady Ziegler, Haley Culbertson, Christopher Goldmann, Marina E. Croy, Noah Willis, Erin Ling, Benjamin Reinhart, Eva C. Lyon

**Affiliations:** 1 Department of Earth and Environmental Geoscience, Washington and Lee University, 204 W. Washington Street, Lexington, VA 24450 USA; 2 Department of Geosciences, Trinity University, 1 Trinity Pl, San Antonio, TX 78212 USA; 3 Geology Department, Whitman College, 345 Boyer Ave, Walla Walla, WA 99362 USA; 4 Department of Biological Systems Engineering, Virginia Tech, Blacksburg, VA 24061 USA; 5 Argonne National Laboratory, Chicago, IL 60439 USA; 6 Present Address: Department of Geological Sciences, Ohio University, Athens, OH 45701 USA

**Keywords:** Mn, Karst, Springs, Well water, Water quality, Virginia

## Abstract

Manganese (Mn) is of particular concern in groundwater, as low-level chronic exposure to aqueous Mn concentrations in drinking water can result in a variety of health and neurodevelopmental effects. Much of the global population relies on drinking water sourced from karst aquifers. Thus, we seek to assess the relative risk of Mn contamination in karst by investigating the Shenandoah Valley, VA region, as it is underlain by both karst and non-karst aquifers and much of the population relies on water wells and spring water. Water and soil samples were collected throughout the Shenandoah Valley, to supplement pre-existing well water and spring data from the National Water Information System and the Virginia Household Water Quality Program, totaling 1815 wells and 119 springs. Soils were analyzed using X-ray fluorescence and Mn K-Edge X-ray absorption near-edge structure spectroscopy. Factors such as soil type, soil geochemistry, and aquifer lithology were linked with each location to determine if correlations exist with aqueous Mn concentrations. Analyzing the distribution of Mn in drinking water sources suggests that water wells and springs within karst aquifers are preferable with respect to chronic Mn exposure, with < 4.9% of wells and springs in dolostone and limestone aquifers exceeding 100 ppb Mn, while sandstone and shale aquifers have a heightened risk, with > 20% of wells exceeding 100 ppb Mn. The geochemistry of associated soils and spatial relationships to various hydrologic and geologic features indicates that water interactions with aquifer lithology and soils contribute to aqueous Mn concentrations. Relationships between aqueous Mn in spring waters and Mn in soils indicate that increasing aqueous Mn is correlated with decreasing soil Mn(IV). These results point to redox conditions exerting a dominant control on Mn in this region.

**Supplementary Information:**

The online version contains supplementary material available at 10.1007/s10653-024-01987-4.

## Introduction

An increasing number of studies have found that Mn in drinking water is a human health concern associated with wide ranging negative effects on IQ and academic performance (Bjørklund et al., 2017; Bouchard et al., 2011; Khan et al., 2012), hyperactivity and attention-deficit hyperactivity disorder (Bouchard et al., 2007; Schullehner et al., 2020), mortality rates (Hafeman et al., 2007; Spangler & Reid, 2010; Spangler & Spangler, 2009), cancer rates (Spangler & Reid, 2010), and more (Corrales Vargas et al., 2022; Langley et al., 2015; Sanders et al., 2014). In light of such research connecting Mn to negative health effects, the World Health Organization recently set a provisional guideline for Mn in drinking water to 80 ppb (World health Organization, 2021). As Mn redox state largely controls its mobility, with suboxic and anoxic systems promoting aqueous Mn(II) and oxic conditions leading to the formation of solid Mn(III) and Mn(IV) (oxyhydr)oxides, drinking water systems that rely on well water are of more concern with regard to elevated Mn than those relying on oxic surface water sources. Drinking water sourced from water wells and springs is particularly prevalent in communities on karst terrain, with an estimated 9–20% of the global population relying on groundwater from karst aquifers (Ford & Williams, 2007; Stevanović, 2019). Thus, understanding Mn (and associated contaminant) mobility in karst aquifers is of global importance.

Drinking water sourced from karst carbonate aquifers may behave differently with respect to aqueous Mn concentrations than those from other aquifer types due to differences in their geochemistry. For example, groundwaters derived from carbonate aquifers within the United States are generally oxic (McMahon & Chapelle, 2008), likely due to increased connectivity between groundwater and surface water in karst terrains, and thus often explains the observed the oxidation of aqueous Mn(II) forming Mn(III/IV) (oxyhydr)oxide minerals in karst aquifers and cave systems (Frierdich & Catalano, 2012; Vesper et al., 2003). These Mn(III/IV) (oxyhydr)oxide minerals are more stable under oxic and basic conditions, and thus their formation could be additionally promoted by karst aquifers due to elevated aqueous bicarbonate concentrations derived from the carbonate source rock, resulting in increased pH. Even if the carbonate aquifer remains suboxic or anoxic, these elevated bicarbonate concentrations can promote the formation of a variety of carbonate minerals (Langmuir, 1971), including the Mn(II) carbonate mineral rhodochrosite (Duckworth & Martin, 2004), which could result in lower aqueous Mn concentrations in karst terrains. However, if Mn contamination is the result of anthropogenic sources, such as the application of Mn-bearing fungicides like manganese ethylenebisdithiocarbamate (maneb) or vehicle emissions via methylcyclopentadienyl manganese tricarbonyl (MMT) (Lytle et al., 1995), karst terrain may lead to an increase in groundwater, soil, and sediment contaminant concentrations relative to other aquifer rock types. Such a scenario has been previously observed for metal pollution (Du Preez et al., 2016; Tao et al., 2020), fertilizers (Jiang et al., 2009; Katz et al., 2009; Panno et al., 2001), and emerging organic contaminants such as pharmaceuticals, per- and polyfluoroalkyl substances (PFAS), phthalates, and microplastics (Lukač Reberski et al., 2022; Mahler & Musgrove, 2019).

As part of the Valley and Ridge province, the Shenandoah Valley is underlain by a variety of Paleozoic strata, including several carbonate units. Thus, the Shenandoah Valley serves as a fertile testing ground for studying Mn contamination in both karst and non-karst aquifers. A recent study investigating Mn contamination in groundwater wells across the United States determined ~ 8% of groundwater wells in Virginia (out of 872 analyzed) have Mn concentrations ≥ 300 ppb (McMahon et al., 2018a, 2018b), the lifetime chronic exposure health advisory limit for Mn set by the Environmental Protection Agency (U.S. Environmental Protection Agency, 2004). It has been argued that this health advisory limit is set too high (Ljung & Vahter, 2007), with chronic exposure to just ≥ 100 ppb Mn in drinking water leading to some health and developmental effects (Langley et al., 2015; Sanders et al., 2014). Approximately 21% of groundwater wells in Virginia analyzed by McMahon et al., (2018a, 2018b) have Mn concentrations exceeding that 100 ppb threshold. With a large percentage of households in Virginia relying on private water wells and springs for their water supply, particularly in rural areas (Benham et al., 2016), Mn may negatively impact the health of tens of thousands of Virginians.

Mn contaminated water wells have previously been reported in several locations throughout Appalachia (Gillispie et al., 2016; Kiracofe et al., 2017; McMahon et al., 2018a; Siegel et al., 2022), including the northeastern portion of the Shenandoah Valley in Virginia (McMahon et al., 2018b). Potential sources of Mn and trace metal contamination in Virginia groundwater and spring water include: dissolution of potential Mn-bearing minerals within the aquifer, the chemical weathering of sediments, and Mn mobilization via desorption from mineral surfaces in the soil–water interface. Recent research on Mn contamination in the Roanoke River (VA) watershed points to mobilization of Mn associated with marbles (Kiracofe et al., 2017). Two other recent studies investigating Mn groundwater contamination, one in the Piedmont region of North Carolina (Gillispie et al., 2016) and one surveying the entire United States (McMahon et al., 2018a) both found that soil geochemistry is strongly linked to Mn concentrations in groundwater. Mn in drinking water is an issue affecting communities across the globe, in regions with vastly different lithologies, soils, and water access (Aiken et al., 2023; Bacquart et al., 2015; Brindha et al., 2020; de Meyer et al., 2017, 2023; Erickson et al., 2019, 2021; Friedman et al., 2023; Kousa et al., 2021, 2022; McMahon et al., 2018a; Podgorski et al., 2022; Sankar et al., 2014; Zhao et al., 2023). To effectively anticipate the risk of Mn (or other trace metal) contamination in drinking water, it is essential to identify the primary factors influencing their concentration levels.

With this research, we investigated aqueous Mn concentrations and other major elements in the aqueous phase in a series of springs and water wells across the Shenandoah Valley region of Virginia. We studied these values alongside soil geochemistry, average Mn redox state in associated soils via Mn K-edge X-ray absorption near-edge structure (XANES) spectroscopy, primary aquifer rock types, and land use. This region was selected because of the high concentrations of Mn in several wells (McMahon et al., 2018a), variable land use practices throughout the region, and reliance on private drinking water sources for a substantial portion of the population. This work seeks to better understand the controls of Mn and other trace metals in waters with dynamic redox conditions, such as springs, and identify the dominant controls on Mn in drinking waters in this region.

## Study area

The Shenandoah Valley is located in the Valley and Ridge physiographic province of Virginia, USA. For our purposes, it is defined not as the hydrographic entity, nor the cultural entity, but rather the structural geologic feature that is also often referred to as the Great Valley. Approximately 150 miles long, it is primarily composed of interbedded Paleozoic sedimentary rocks, including limestones, dolomites, shales, quartz arenites and other sandstones (Fig. S1), as well as conglomerates, siltstones, mudstones, and coals (Virginia Division of Mineral Resources, 1993). The folding and faulting of the late Paleozoic Alleghenian Orogeny were responsible for uplifting and exposing these rock types across the region (e.g., Hatcher et al., 1989; Rast, 1984). Subsequently, the Shenandoah Valley formed as a result of the differential weathering of chemically susceptible limestones and shales, whereas the adjacent ridges are composed of more resistant sandstones and, in the case of the Blue Ridge bounding to the East, crystalline igneous and metamorphic rocks (Fig. S1). The Massanutten Synclinorium underlies much of the Shenandoah Valley, with Massanutten Mountain (a sandstone dominated ridge) running northeast through the uppermost 1/3 of the Valley, splitting the Shenandoah River into the North and South forks on either side of the Mountain. Prominent thrust faults separate the Shenandoah Valley from the Blue Ridge, and from other prominent ridges to the west. These faults can serve as important conduits for the flow of groundwater.

**Fig. 1:**
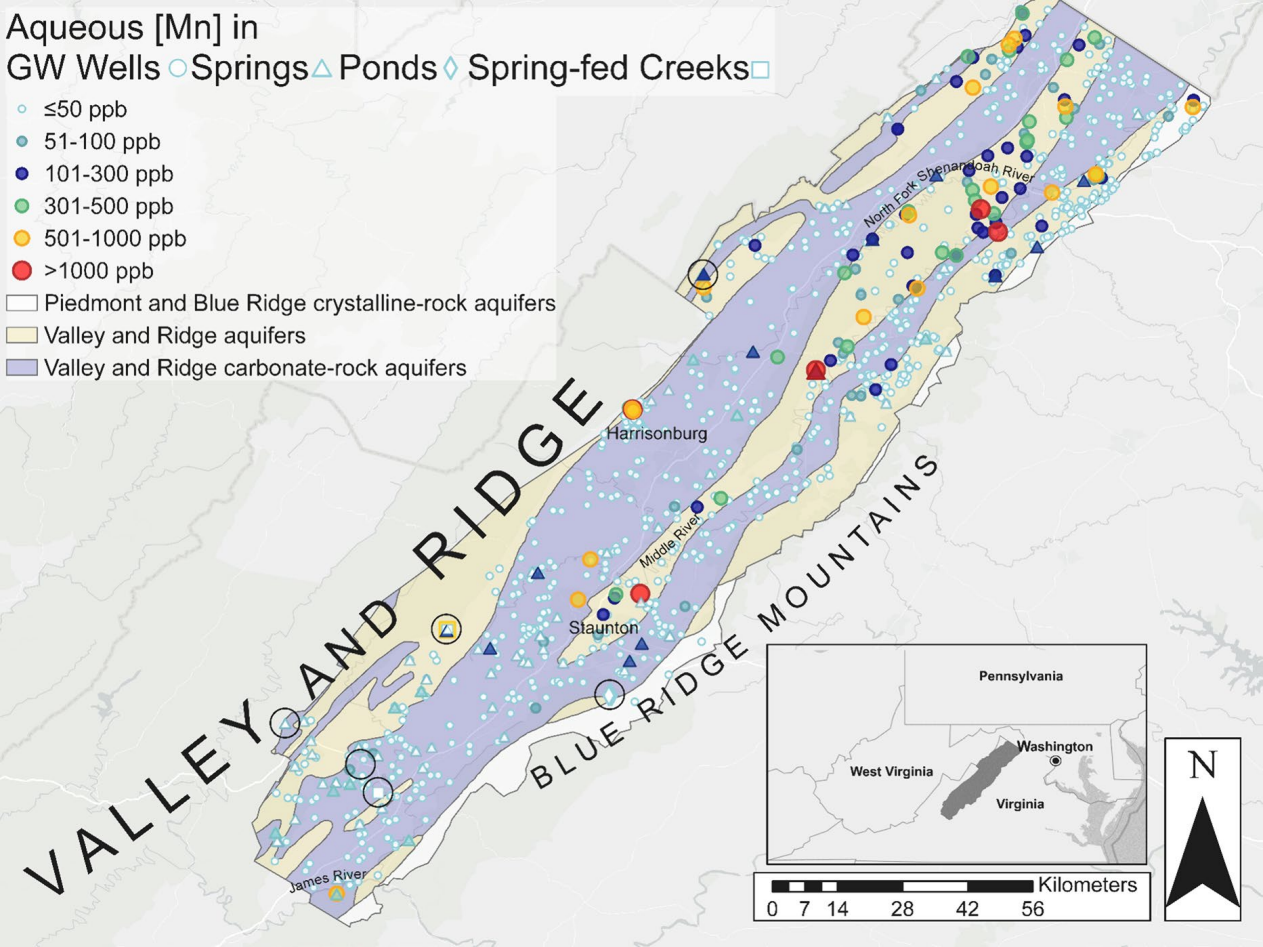
Spatial distribution of sampling sites for springs (triangles) and private drinking water sources from the VAHWQP data set and the USGS groundwater well network (circles), as well as ponds (diamonds), and spring fed creeks (squares) overlying the major aquifer types of Valley and Ridge aquifers (yellow), Valley and Ridge carbonate rock aquifers (purple), and Piedmont and Blue Ridge crystalline-rock aquifers (white). Field locations (each representing an area in which multiple field samples were collected) are highlighted by the clear circles with black outlines. Sites are color coded by [Mn]_aq_, with values below the threshold of concern for chronic aqueous Mn exposure of < 50 ppb (white outlined in blue), 51–100 ppb (light blue), those between 101–300 ppb (dark blue), 301–500 ppb (green), 501–1000 ppb (yellow), and > 1000 ppb (red)

The principal aquifers in this region are siliciclastic and carbonate aquifers (Trapp & Horn, 1997), so termed “Valley and Ridge aquifers” and “Valley and Ridge carbonate-aquifers” with some crystalline-rock aquifers along the easternmost portion of the Valley at the base of the Blue Ridge escarpment (Fig. 1). The substantial folding within the Valley and Ridge province can make delineating aquifer extent difficult, but aquifer boundaries generally align with lithologic unit boundaries (Miller, 1990). Carbonate and sandstone aquifers are the most common in the Great Valley region of the Valley and Ridge province (Fig. 1), with carbonate aquifers occurring within valley floors and siliciclastic aquifers along ridges (Trapp & Horn, 1997). The carbonate aquifers can be quite productive; their chemical dissolution features and fractures lead to fast rates of groundwater conduit flow and high water yields (Trapp & Horn, 1997). Groundwater in the Shenandoah Valley typically flows along faults, fractures, bedding planes, or through dissolution features, and often emerges as springs or into streams where the landscape meets the water table (Trapp & Horn, 1997).

Thick soil layers have developed from the weathering of limestone bedrock in the Shenandoah Valley (Ciolkosz et al., 1989). Thus, the Shenandoah Valley and other smaller valleys within the Valley and Ridge province have been able (and continue) to support large agricultural communities (Marschner, 1940). These soils are high in clay and Fe oxide content, both of which can strongly bind trace metals (including Mn). Mn may also occur in small amounts as Mn oxides (which also act as strong trace metal sorbents), with Mn occurring naturally in most soils (U.S. Environmental Protection Agency, 2004). Many soils in the broader region experienced increased weathering rates as a result of historic tilling, resulting in increased soil weathering and erosion rates (Costa, 1975; Dotterweich et al., 2014; Hall, 1937; Trimble, 2008), likely expediting mineral dissolution processes.

Major soil map units within the Shenandoah Valley, VA region are, as expected, comprised of several soil series within each map unit (Fig. S2) reflecting mixtures of inceptisols, alfisols, and ultisols (Soil Survey Staff, 2023). Ultisols are the dominant soil order within the valley floors due to their age and highly weathered nature, while alfisols are primarily found within forested areas. Both alfisols and ultisols can contain relatively high clay content, but alfisols are typically more productive than ultisols due to the higher degree of weathering and leaching ultisols experience (Soil Survey Staff, 1999). Meanwhile, inceptisols represent the least weathered of the three primary soil orders in the region (Soil Survey Staff, 1999), and are more commonly found on slopes and ridges within the Shenandoah Valley, VA region. While these three soil orders can be conceived of occurring in different regions with respect to valleys and ridges, the dominant soil map units within the Shenandoah Valley are more often mixtures of the primary soil orders. For example, the Frederick-Carbo unit reflects a mixed ultisol (Frederick) and alfisol (Carbo), while Litz-Groseclose is a mixed inceptisol (Litz) and ultisol (Groseclose), and the Mysersville-Catoctin series is an alfisol (Myersville) inceptisol (Catoctin) mixture.

Another potential source of soil Mn is the historic mining of Fe, Mn, and other metal ores. Portions of the Shenandoah Valley have historically been home to metal mining, including Fe, Mn, tin, cobalt, copper, and nickel (Knechtel, 1943). Mn ore deposits are thought to have formed via mobilization of Mn via water–rock interactions, precipitating out in breccias and along faults (Carmichael et al., 2017). Mn enrichment in soils near Mn ore mines has been previously observed (Jordan et al., 2019). Trace metal contamination of nearby waters may occur from ore mining by disturbing the surrounding soils and sediments, increasing weathering rates and inducing changes in soil, sediment, and/or water geochemistry that enhances metal mobilization to the aqueous phase (Black et al., 2004; Kossof et al., 2012; Miller, 1997).

## Materials and methods

### Spring and well water database compilation

For this research we collected and analyzed spring waters to add to the already established well and spring water databases [via the USGS National Water Information System (NWIS) and the Virginia Household Water Quality Program (VAHWQP; www.wellwater.bse.vt.edu)]. The VAHWQP and USGS NWIS data sets were combined to ultimately yield 1,815 well samples and 91 spring samples, after removing duplicates (retaining the most recently collected data in the case of duplicate samples for the same location, water source, treatment, and depth). Samples from the USGS NWIS used in this study were obtained from the compilation made available by McMahon et al. (2018b). Samples in this data set were collected from 1988 to 2017 and were filtered (0.45 µm; acrylic polymer or glass fiber filter membranes) and analyzed for NO_3_ (as N) and sulfate, with filtered samples additionally acidified prior to analysis for Fe and Mn concentrations. Dissolved oxygen, pH, and DOC were also analyzed following protocols outlined by McMahon et al., 2018a, 2018b. Common assessment levels for Mn, Fe, sulfate, nitrate, and DOC are reported as 4 ppb, 10 ppb, 1 ppm, 0.1 ppm, and 0.23 ppm, respectively (McMahon et al., 2018b). Additional information, such as water source type and well depth were also included in the USGS NWIS data set and included in data analysis for this current study.

Samples from the VAHWQP, all from private residences, were collected from tap water systems after flushing for five minutes between the months of February–November from 2009 to 2021. The use of water treatment systems (e.g., water softeners, sediment filters, UV light filters, iron removal filters, carbon filters, acid neutralizers, and chlorinators), well depth, and recent household illnesses were self-reported. All water samples (filtered if noticeable particles present; 0.45 µm filter) were analyzed via ion chromatography for NO_3_ (as N) and fluoride and acidified samples (to 2% nitric acid using concentrated trace metal grade nitric acid) via ICP-MS for 29 trace and major element aqueous concentrations (Li, Na, Mg, Al, Si, P, S, Cl, K, Ca, Ti, V, Cr, Fe, Mn, Co, Ni, Cu, Zn, As, Se, Sr, Mo, Ag, Cd, Sn, Ba, Pb, and U) via inductively coupled plasma-mass spectrometry (ICP-MS; Thermo Electron iCAP RQ). A series of 8 standards were used to generate ICP-MS calibrations, with a standard and blank included between every ten samples. If measured values were found to be below detection limit, they were reported as being half the detection limit (Reimann, 2008), with a detection limit of 0.1 ppm for NO_3_^−^ and F^−^ and of 1 ppb for element concentrations measured via ICP-MS.

It is important to note that no springs from sandstone aquifers are in this compiled data set, both in the springs sampled for this current study and in the VAHWQP and USGS data sets for the Shenandoah Valley region. Several other studies focusing on springs in the Valley and Ridge region also exhibit a similar bias toward more carbonate springs than sandstone (Johnson et al., 2011; McColloch, 1986; Saad & Hippe, 1990). The lack of sandstone springs included in this current study and in previous studies is partly due to access issues and partly the relative paucity of sandstone-originating springs in the Valley [being relegated primarily to ridge lines (Fig. S1)], an issue discussed thoroughly in Vesper and Herman (2020). Additional limitations of the current study include the wide range of years over which samples were collected (therefore the results are not representative of one specific moment in time), the lack of samples from winter months in the largest data set, and the variability in parameters measured between data sets (e.g., both data sets lack all the measurements required to generate ionic balance calculations).

### Sample collection and field analyses

Twenty-three springs and seeps were selected for sampling during June–August of 2021 based on accessibility, landowner permission, and regions of interest based on a preliminary analysis of the private drinking water data set from the Virginia Household Water Quality Project (VAHWQP). These sites include several springs and spring-fed creeks in the Augusta Springs and Wetlands area (ASW), Kerr’s Creek (KC) area, Bubbling Springs, and Orkney Springs. In addition, 5 meteoric ponds and 1 likely spring-fed pond within a sinkhole pond complex known as Maple Flats were also sampled and analyzed to help differentiate between soil–water interactions and groundwater inputs. The Maple Flats Complex, located within the George Washington and Jefferson National Forests, are a series of clay bottom ponds that exhibit unpredictable ephemeral drying and filling behavior, with the exception of one pond that has never been observed to go dry and is largely considered to be spring fed, Spring Pond (Fleming & Alstine, 1999). All ponds at the Maple Flats Pond Complex contained water at the time of sampling (August 2021).

Water, soil, and in-situ field measurements were collected at all 30 sites. YSI field probes were used to measure in-situ water pH, dissolved oxygen (DO), temperature, and specific conductivity. Water samples were collected at the same depth as in-situ measurements in acid washed polypropylene bottles. These water samples were stored in sealed 250 mL polypropylene bottles (unfiltered samples for long term sample storage) and 50 mL polypropylene centrifuge tubes (filtered samples for laboratory analyses as described below; 0.2 μm mixed cellulose esters membranes; Millipore ®) in a cooler prior to transport back to the laboratory for further analyses, as described in Sect. 2.3. Upon return to the laboratory, an aliquot of the filtered water samples was acidified to 2% nitric acid (trace metal grade; Fisher ®) for subsequent trace metals analysis.

Soil samples from 0 to ~ 30 cm depth were collected from each site as close as possible to each spring, with additional cores collected up- and down-topographic gradient of the springs. Soil cores were collected using a stainless steel soil coring device (AMS ®) and stored wrapped within polyethylene sampling bags to retain general core structures. Those from reducing environments containing gley soil susceptible to rapid oxidation kinetics were vacuum sealed immediately post sampling to slow oxidation. All samples were transported from the field to the laboratory in a cooler within 6 h, at which point they were immediately refrigerated (4 °C).

### Laboratory analyses

Water geochemistry of field samples collected from springs, seeps, and ponds were analyzed via multiple methods to gain a comprehensive understanding of water geochemistry at each site. Filtered and acidified water samples were analyzed for aqueous concentrations via ICP-MS for the 28 trace and major elements listed above in Sect. 2.2. Filtered samples were analyzed for major cation and anion concentrations (Ca^2+^, Mg^2+^, K^+^, NH_4_^+^, Na^+^, Li^+^, PO_4_^3−^, NO_3_^−^, SO_4_^2−^, Br^−^, NO_2_^−^, Cl^−^, F^−^) by ion chromatography (IC), using a series of 7 standards and calibration blanks, with standards and blanks interspersed every ten samples throughout the run. Alkalinity of refrigerated water samples was determined using Hach Alkalinity Titration kits following the bromocresol green-methyl red titration method, usually within hours of returning from the field.

For soils, refrigerated soil cores were separated into different horizons based on Munsell color designations. A designated “top” and “bottom” for each core was assigned to each core by either distinguishable color differences in horizon for the uppermost horizon (“top”) and the bottommost horizon (labeled “bottom”) or at least 3 cm from the respective end of the core. Each Munsell-defined soil horizon was subsampled for analyses requiring dried soil samples (105 °C). Total Mn and Fe concentrations were determined on dried and ground (agate mortar and pestle, ~ 8 min) soil samples using X-ray fluorescence (XRF; Thermo Scientific Niton XL3t). The USGS standards Green River Shale (SGR-1b), Guano Valley Andesite (AGV-2), and Columbia River Basalt (BCR-2) were used to calibrate the XRF for Mn and Fe, with calibrations performed with every set of five sample analyses. Samples with reported values below the limit of detection (< 84 ppm Mn), representing over 50% of the data set, were excluded from analyses. Uncertainties associated with XRF measurements are conservatively estimated to be ~ 7–10% based on XRF measurements of standards as samples during sample runs.

Dried samples were also analyzed for mineralogy by X-ray diffraction (XRD; Diano 2100-E instrument with a copper target). Information on soil particle morphology and elemental composition was obtained by scanning electron microscopy paired with energy dispersive X-ray spectroscopy (SEM–EDS) on uncoated, air dried samples (Zeiss EVO MA 15 SEM paired with an Oxford EDS X-Max), operated under variable pressure (Zeiss EVO VPSE G3). Soil moisture content was determined via gravimetric analysis on 3–5 g soil drying to 105 °C. These same soils were also analyzed for percent organic matter via loss on ignition to 375 °C at a rate of 5 °C per minute. Soil pH was determined by measuring the supernatant pH of a 1:2 mixture of soil:0.1 M calcium chloride (CaCl_2_) solution (Sumner, 1994).

### XANES spectra collection and analysis

Soil samples deemed to have adequate amounts of soil Mn to allow for Mn K-edge XAS were analyzed by Mn K-edge X-ray absorption near edge structure (XANES) spectroscopy (note: XRF analyses were only able to be obtained after the allocated time for XAS analyses, thus the initial screening for adequate soil Mn was conducted via SEM–EDS). These XANES spectral samples were prepared from soil wet pastes (to prevent alteration of Mn speciation upon drying) and mounted in polycarbonate sample holders, protected with 25 µm Kapton film and sealed with 25 µm Kapton tape. A suite of solid and shelf-stable Mn oxidation state standards was also prepared by grinding the solids with an agate mortar and pestle (~ 8 min) and sealing with Scotch tape following previous protocols for Mn oxidation state standard preparation (Hinkle et al., 2023). XANES spectra of soil samples and standards were collected at Argonne National Laboratory’s Advanced Photon Source at beamline 12-BM. The Si(1 1 1) fixed-offset double-crystal monochromator was detuned by 60% and calibrated to the Mn K edge (6539 eV) with a Mn foil. A 7-element fluorescence detector (Vortex ME7) was used to collect Mn K-edge spectra of samples.

Initial data processing of XANES spectra (averaging scans and normalizing spectra) was conducted using Athena (Ravel & Newville, 2005), a graphical user interface for operating IFEFFIT (Newville, 2001). Athena was also used to calculate the average Mn oxidation state (AMOS) for select soil samples via linear combination fits (LCFs) of the Mn K-edge XANES spectra collected of the Mn oxidation state standards (Mn(II): rhodochrosite, manganese chloride, manganese sulfate; Mn(II/III): hausmannite; Mn(III): manganite, feitknechtite, bixbyite; and four Mn(IV) standards from Manceau et al. (2012): pyrolusite, ramsdellite, KBi (a K^+^-birnessite), and Ca_2_Mn_3_O_8_) (Figure S3). These LCFs, following a modified protocol derived from Manceau et al. (2012) in which fits are constrained to non-negative loadings of six or fewer oxidation state standards, yield fractions of Mn(II), Mn(III), and Mn(IV). These fractions, along with the total [Mn]_solid_ in each soil derived by XRF analyses yield the concentration of solid-associated Mn(II), Mn(III), and Mn(IV) in each soil sample.

### Geospatial and statistical analyses

Spatially linking the data from each field site and sampling site from the spring and well water databases to potentially relevant factors (such as aquifer type, most likely aquifer rock type, soil type, distance to nearest surface water body, distance to nearest mafic dike, sill, or fault, etc.) was completed using ArcGIS Pro and the spatial join and near features, as appropriate. Springs were included in the analysis regarding distance to the nearest surface water body (acknowledging the fact their source can be far from their emergence), as previous studies have found that karst springs can be recharged by surface waters (Jeelani et al., 1998; Miller et al., 2015). Thus, nearby surface water may impact groundwater and spring water geochemistry. The substantial folding within the Valley and Ridge can complicate correctly identifying the lithology of groundwater aquifers in any one location. Generally, aquifer boundaries align with surface lithology (Miller, 1990), and thus pairing sampling location with lithology yields the “most likely” aquifer for any one well or spring. This approach allows for the relationships between Mn concentrations and these potentially relevant factors to be assessed, in addition to the relationships between Mn concentrations and other geochemical data collected for each site.

However, Mn concentrations in wells and springs were not normally distributed, so non-parametric analyses were used for statistical comparisons. To compare Mn concentrations in the various aquifer lithologies, two separate Kruskal–Wallis tests with Bonferroni-adjusted significance thresholds (α = 0.05) were used for well Mn concentrations and spring Mn concentrations. Then Wilcoxon signed rank tests (α = 0.05) were used to compare lithologies to identify those that had statistically different Mn concentrations. A separate Wilcoxon signed rank test was used to compare Mn concentrations in wells and springs based on whether Fe was greater than or less than 1 mol % of the sum of Fe, Si, and Mg concentrations.

## Results

### Spring and well water aqueous geochemistry

#### Aqueous Mn concentrations by aquifers

Mn concentrations in well waters range from below detection limit to 1926 ppb Mn, with an average of 37 ppb Mn (*n* = *1,815*). Meanwhile, springs have generally lower Mn concentrations, with an average of 32 ppb, ranging from below detection limit up to 1348 ppb Mn (*n* = *119*). Springs and well waters exhibit a similar spatial distribution, with low aqueous Mn in the USGS-designated “Valley and Ridge carbonate-aquifers” and elevated aqueous Mn primarily clustered in the USGS-designated “Valley and Ridge aquifers” (aquifers with primary rocks comprised of non-carbonate bearing lithologies) (Fig. 1).

**Fig. 2:**
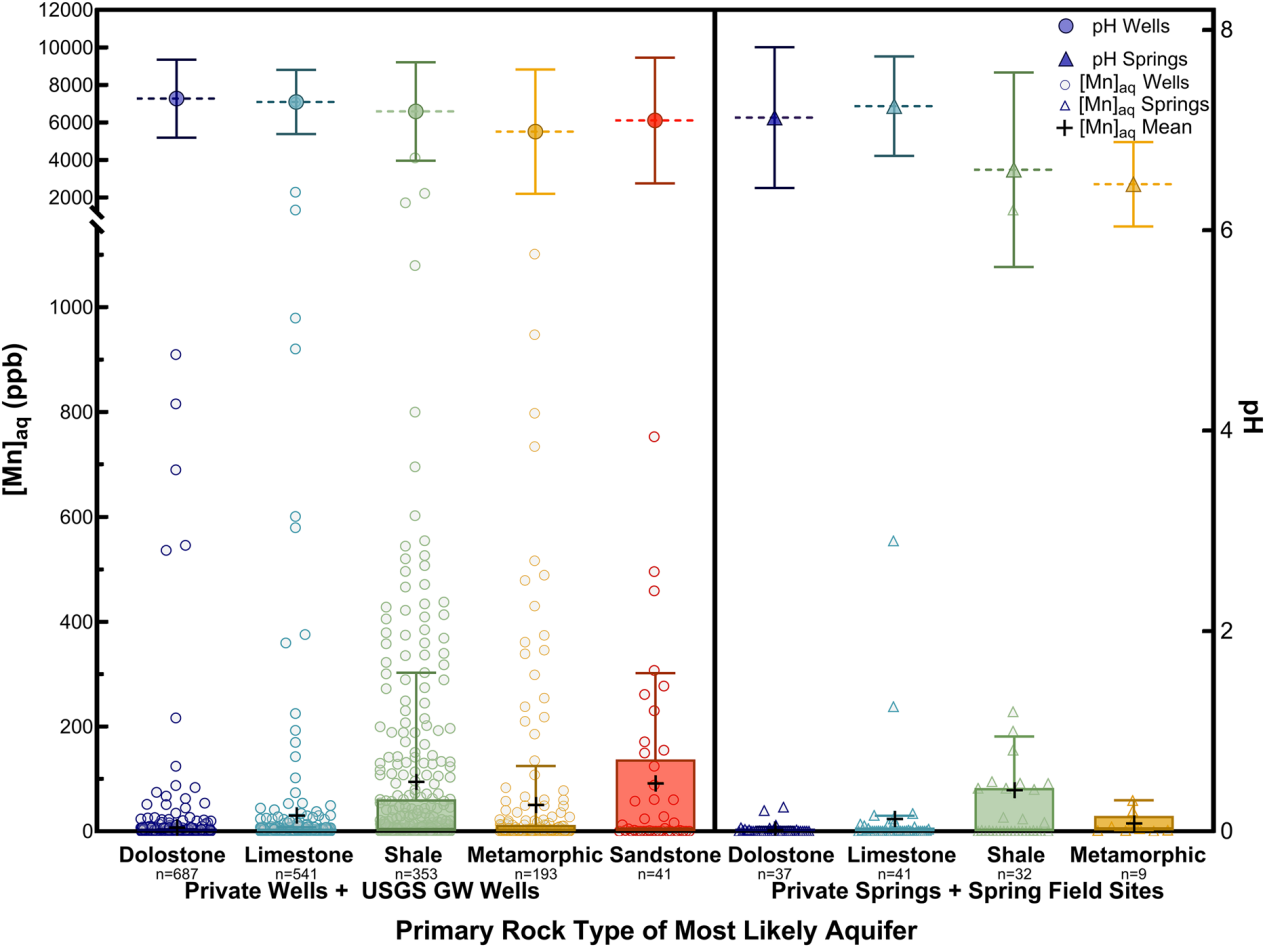
Box plot (25th and 75th percentiles) with whiskers (10th and 90th percentiles) with means denoted (black +), and distribution of Mn concentrations (data points) by the most likely aquifer primary rock type (dolostone as dark blue, limestone as teal, shale as green, metamorphic as yellow, and sandstone as red) for private groundwater well sources within the VAHWQP database and USGS groundwater wells (white circles) and private spring sources within the VAHWQP database and spring field sites (white triangles). Average pH for each rock type and water source is plotted along the secondary y-axis with color corresponding to its rock type as filled in circles (wells) or as filled in triangles (springs) with mean denoted by horizontal dotted lines and 1σ standard deviation as vertical lines. The total number of samples (n) is denoted underneath each aquifer primary rock type label

With these distinct differences between carbonate and non-carbonate Valley and Ridge aquifers within the Shenandoah Valley, average Mn concentrations and pH were determined for the aquifer lithology (Fig. 2). Comparing springs with wells reveals similar patterns for Mn concentrations in dolostone, limestone, and shale aquifers regardless of water source (Fig. 2). Due to an abundance of samples below detection limit, the median for each data set is very low (often below detection limit) (Table 1). It should be noted the data does not fit a log-normal distribution or a Gaussian distribution, and thus it is important to consider a number of statistical parameters to fully describe the data set. The maximum Mn concentrations of springs are consistently lower than those of wells of the same lithology (Fig. 2). Several aquifer rock types for springs even have maximum Mn concentrations well below the 100 ppb threshold, while the maxima for all well water lithologic suites all nearly exceed that by an order of magnitude (Table 1).

**Table 1:** Descriptive statistics of aqueous Mn concentrations in the Shenandoah Valley for water wells and springs by most likely aquifer primary rock types

*Percentiles for aqueous [Mn] (ppb)*								
Aquifer Primary Rock Type	#	Maximum	10th	25th	50th (Median)	75th	90th	% > 100 ppb Mn
*Water Wells*								
***All***	***1815***	***7100***	***BDL***^***a***^	***BDL***	***BDL***	***3.2***	***41.5***	***7.0%***
Dolostone	687	910	BDL	BDL	BDL	1.1	4.0	1.0%
Limestone	541	7100	BDL	BDL	BDL	1.9	7.7	2.6%
Metamorphic	193	1102	BDL	BDL	1.6	12.1	124.6	10.4%
Sandstone	41	753	BDL	BDL	5.1	137.3	301.8	26.8%
Shale	353	4125	BDL	BDL	4.4	61.1	302.5	21.2%
*Springs*								
***All***	***119***	***1348***	***BDL***	***BDL***	***0.6***	***8.7***	***80.2***	***4%***
Dolostone	37	46.3	BDL	BDL	BDL	1.4	8.2	0%
Limestone	41	554.5	BDL	BDL	BDL	7.1	29.6	4.9%
Metamorphic	9	58.7	0.6	1.9	5.6	29.4	59.3	0%
Shale	32	1348	BDL	BDL	1.3	82.5	180.8	9.4%

^a^BDL for ‘below detection limit’ of 1 ppb Mn for samples analyzed by ICP-MS

These observed differences in Mn concentrations by aquifer lithology were determined to be statistically significant. Kruskal–Wallis tests (α = 0.05) for wells and springs yield p-values of < 2 × 10^–16^ and 0.00607, respectively. Wilcoxon tests (α = 0.05) indicate that several aquifers are significantly different from one another (Table S1). This lithologic classification of the primary rock type for the most likely aquifer for each sample reveals that shales and sandstones have the highest percentage of wells above the 100 ppb threshold for chronic exposure to Mn, yielding 21.2% and 26.8%, respectively (Table 1). Many wells originating in metamorphic lithologies do contain relatively high Mn concentrations (Fig. 2), but to a much lower extent than that of shales and sandstones, with 10.4% of metamorphic wells exceeding 100 ppb Mn (Table 1). Metamorphic wells were found to be significantly different in terms of Mn concentrations for all lithologic comparisons, with the exception of sandstone (Table S1). While no sandstone aquifer springs were included in this data set (as explained above in Sect. "Spring and well water database compilation"), springs originating in shale-dominated lithologies were prevalent through the Valley and are the spring type with the highest Mn sampled at an individual site, with 9.4% of shale-aquifer springs exceeding that 100 ppb (half that of wells) (Table 1).

On the other hand, both carbonate aquifer primary rock types, dolostone and limestone, have relatively low Mn concentrations for springs and wells (Fig. 2), with median values even below detection limit (Table 1). However, the second highest Mn concentrations in both the well water and spring suites occurs in limestone lithology (Fig. 2). Meanwhile, dolostone aquifers have both low Mn across nearly all samples, with even the 90th percentile at just 4.0 and 8.2 ppb for dolostone wells and springs, respectively (Table 1). Although some well waters in dolostone aquifers have concentrations > 100 ppb Mn, it only represents 1% of wells (Table 1). Mn in dolostone and limestone aquifer-sourced springs and wells were found to not be statistically different from one another (Table S1), confirming these aquifers are similar with respect to aqueous Mn.

The relatively low Mn concentrations observed in wells and springs in carbonate aquifers in the Shenandoah Valley is unlikely to be explained due to pH effects, as the mean pH for all aquifer rock types is circumneutral, between pH of 6.5–7.3 (Fig. 2). Comparing well water aqueous Mn against pH (Figure S4A) does not demonstrate robust correlations, with only weak correlations for all rock types, and therefore any variation in pH does not appear substantial enough to account for the observed differences in Mn observed across lithologies. For the data set taken as a whole, there is a very weak negative correlation between pH and Mn (r_s_ =  − 0.191, *n* = 1808, *p* < 0.0001), indicating that higher aqueous Mn tends to be weakly correlated with more acidic pH. Unexpectedly, hardness results in a strong positive correlation for wells in shale lithologies (r_s_ = 0.635, *n* = 342, *p* < 0.0001) while all aquifer rock types combined yield a weak positive correlation (r_s_ = 0.245, *n* = 1720, *p* < 0.0001) (Figure S4B). The hardness of shales aligns with that of carbonates [not wholly unexpected due to the calcareous nature of many shales in the Shenandoah Valley (Cardwell et al., 1968)], while shales exhibit typically higher Mn concentrations than carbonate bearing aquifers (Figure S4B).

As expected, dissolved oxygen and Mn exhibit a negative correlation for all rock lithologies and for the data set assessed as a whole (r_s_ = − 0.328, *n* = 84, *p* = 0.0003), with dolostone and limestone yielding the highest median dissolved oxygen values (6.8 and 5.7 mg/L, respectively) of all aquifer rock types (Figure S4C). However, with dissolved oxygen measurements of the wells only available for the USGS data set (representing 84 samples with dissolved oxygen and Mn measurements), and with the vast majority of those samples in limestone (*n* = 40) and dolostone (*n* = 31) aquifers, it is difficult to ascertain the extent to which dissolved oxygen varies by aquifer rock type in the current data set (Figure S4C).

Thus, it is useful to consider proxies for redox conditions in groundwater, such as nitrate (indicative of oxidizing conditions, with *n* = 1807) and aqueous Fe (with > 100 ppb Fe as indicative of reducing conditions (Erickson et al., 2019; McMahon & Chapelle, 2008). Consistent with the dissolved oxygen results, there are negative correlations (ranging from very weak to moderately weak) between nitrate and aqueous Mn (Figure S5A). In terms of proxies for reducing conditions, dolostone and limestone well waters and springs typically have aqueous [Fe] < 100 ppb, with 90th percentiles well below that threshold (Table S2), indicating that these aquifer rock types in well weathered karst terrains are more oxidizing than other, less weathered aquifer rock types, consistent with USGS findings of Valley and Ridge carbonate aquifers being far more oxic than the adjacent Valley and Ridge aquifers (which are primarily siliciclastic in nature) (DeSimone et al., 2014). Positive correlations are present between Fe and Mn for all primary rock types (with r_s_ ranging from 0.370 (limestone) and 0.668 (sandstone), all with *p* < 0.0001) (Figure S5B).

#### *Mn mobilization from aquifers explored *via* ternary analysis*

**Fig. 3:**
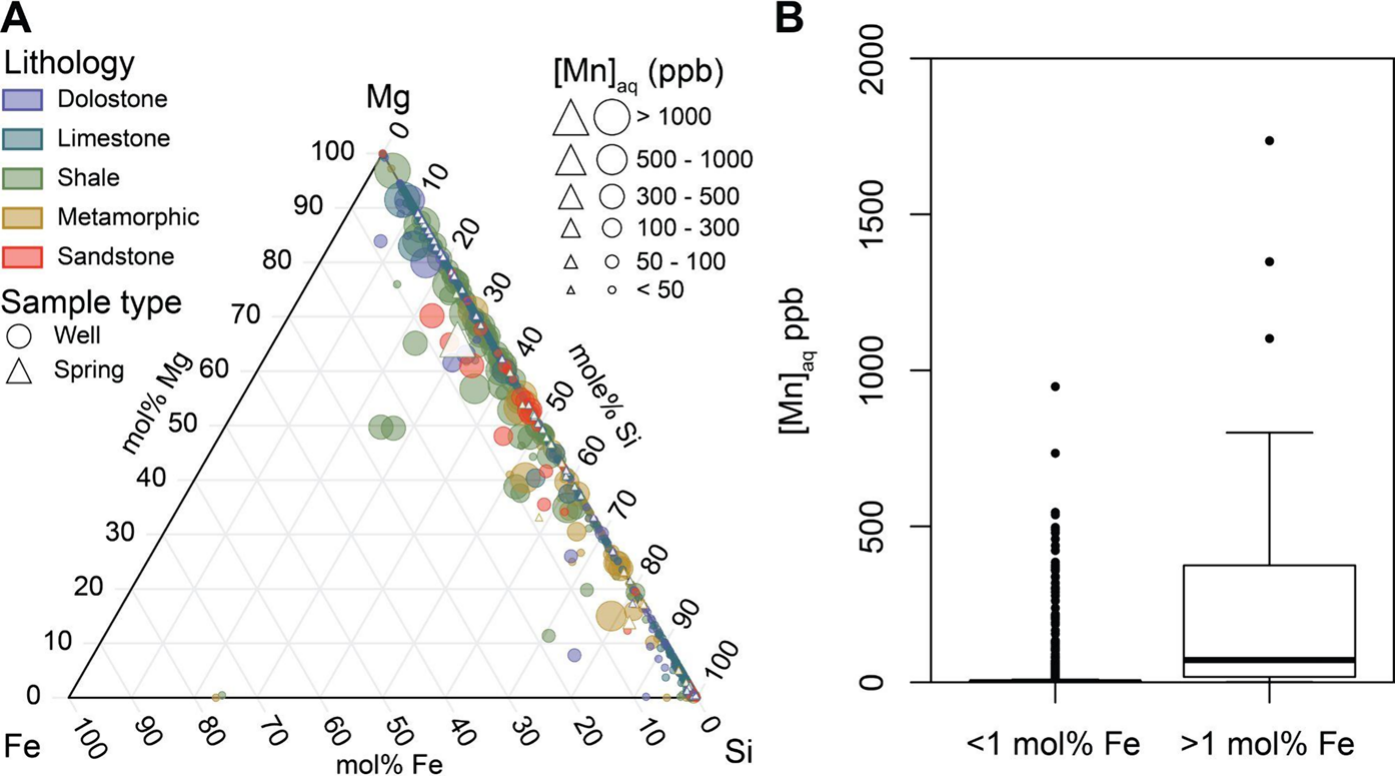
Ternary plot of Fe, Mg, and Si as proxies for reductive dissolution, carbonate weathering, and silicate weathering, respectively. Aqueous Mn concentrations from wells (circles) and springs (triangles) are scaled by concentration and colored by aquifer lithology (**A**). Boxplots of aqueous Mn concentrations for samples in which Fe is < 1 mol% of the Fe/Mg/Si sum vs for samples in which Fe is > 1 mol% of the Fe/Mg/Si sum (**B**)

Mn can be present in minor and trace amounts in a variety of aquifer minerals (silicates, carbonates, oxides). Furthermore, although an aquifer may be designated as a primary rock type, rock formations may be heterogeneous, with a primary rock type and interlayered with secondary rocks and their associated minerals (e.g., a shale with secondary limestones), and Mn in groundwater might originate in minerals other than those found in the primary rock type (i.e., Mn mobilized from secondary carbonates in a primarily shale formation). Ternary analyses of well water chemistry compared the mole fractions of Fe, Mg, and Si as endmember proxies for reduction of hydroxides, carbonate dissolution, and silicate weathering, respectively, to better determine mineral weathering reactions contributing to Mn mobilization. As expected, the carbonate aquifers (limestone and dolostone) exhibit higher proportions of Mg, while sandstones and metamorphic rocks display elevated fractions of Si (Fig. 3). Meanwhile, shales are more evenly distributed between Mg and Si (Fig. 3). Relatively high proportions of Fe occurred sporadically in each rock type, suggesting hydroxide reduction is not specific to any one rock type, although it does occur more commonly in shales.

The correlation between Fe and Mn is clear, with the majority of samples < 50 ppb aqueous Mn plotting near 0 mol% Fe (with an average mol% Fe of just 0.19 mol%), and increasing aqueous Mn as the mol% Fe increases (Fig. 3). Notably, shales demonstrate the highest concentrations of both Fe and Mn. For samples with aqueous Mn > 50 ppb, the average mol% Fe is 2.0 mol%. Conversely, the aqueous Mn is significantly higher (Wilcoxon signed-rank test *p* < 2 × 10^–16^) for samples with Fe > 1 mol% (with a median a of 71.9 ppb) compared to samples with Fe < 1 mol% (with a median Mn of 0.6 ppb). Furthermore, 44% of samples with Fe > 1 mol% were above 100 ppb, whereas only 6.7% of samples exceeded 100 ppb Mn if Fe < 1 mol% (Fig. 3).

The observed positive correlation between Mg and Mn (Fig. 3) is revealed to be most strongly correlated for the shale primary aquifer rock group (r_s_ = 0.697, *n* = 342, *p* < 0.0001) and for sandstones (r_s_ = 0.592, *n* = 40, *p* =  < 0.0001) (Figure S5C). With important geologic formations in the Shenandoah Valley region consisting of both calcareous shales and calcareous sandstones (e.g., Waynesboro Formation), it is possible the observed positive correlations are the result of shale and sandstone dissolution, thereby mobilizing solid-associated Mg and Mn. We see nearly identical behavior with respect to Ca and Mg (Figure S5D), yet only weakly positive to weakly negative correlations between Si and Mn for shales and sandstones, respectively (Figure S5E). Shales and sandstone weathering should result in increased aqueous Si, thus if the correlations observed for Mg, Ca, and Mn are attributable to shale and sandstone aquifer rock dissolution, it is likely due to the calcareous fraction dissolving rather than the whole rock, suggesting that Mn in these calcareous shale and sandstone aquifers is associated with the calcareous portion.

#### Aqueous Mn and depth profiles

Because the exposure of surface lithology is largely the product of differential weathering in the Shenandoah Valley, and lithology may control aspects such as well depths, it is prudent to investigate the relationship between well depths and aqueous Mn concentrations. As shown in Figure S6, the more shallow the well, the more elevated the aqueous Mn, consistent with previous research on Mn in groundwater wells across the United States (McMahon et al., 2018a). Wells deeper than 250 m are all far below the 100 ppb Mn threshold, regardless of rock type (Figure S6). However, it should be noted that the wells with depths > 250 m, none are sandstones or shales, suggesting a potentially lithologic component to explain the well depth—Mn observations, consistent with the results from the ternary analysis discussed above.

In fact, comparing well depths with the aqueous concentrations of other major elements of interest reveals lithologic trends for each suite of elements. The distribution of aqueous Fe concentrations [with aqueous [Fe] > 100 ppb as an indicator of reducing conditions (Erickson et al., 2019; McMahon & Chapelle, 2008)] with respect to well depth exhibits a similar trend to that of Mn, albeit with increased scatter (Figure S6). Dolostone and limestone aquifers tend to have lower aqueous Fe concentrations, while shales tend to have higher aqueous Fe (Figure S6). Intriguingly, four wells in dolostone aquifers with low aqueous Mn concentrations (< 50 ppb) have water geochemistry indicative of reducing conditions (note: no dissolved oxygen measurements are available for these samples), with nitrate concentrations below detection limit and very high Fe concentrations (> 2 mg/L) (Erickson et al., 2019; McMahon & Chapelle, 2008), suggesting that additional processes beyond just redox reactions can impact groundwaters sourced from dolostone aquifers.

Considering aqueous [Si] (common in waters associated with sandstone aquifers) as a function of well depths reveals that aquifers situated in metamorphic, sandstone, and shale formations tend to exhibit higher Si concentrations (consistent with the ternary analysis), with wells with the highest measured [Si] predominantly located at depths ranging from ~ 50–150 m (Figure S6). As expected, wells with highest aqueous [Mg] (as a major ion in carbonate aquifers) are found in limestone and dolostone wells, with some instances of high Mg in shales (Figure S6), consistent with the presence of calcareous shales within the Shenandoah Valley (Cardwell et al., 1968). The distribution of Mg across well depths is fairly scattered, however there is a notable peak in high Mg concentrations at depths of 200–300 m (Figure S6). With deeper wells at 200–300 m composed of more Mg-rich and carbonate bearing rock types such as dolostone, it is thus plausible that the interconnected relationship between well depths and lithology may partly explain the primary aquifer rock type trends observed for aqueous Mn. However, Figure S5 also demonstrates that elevated Mg concentrations occur at shallow and deep well depths. With many shallow wells in dolostone bearing aquifers exhibiting high aqueous Mg but low Mn, while many shallow wells in limestone and shale aquifers exhibit high aqueous Mg and high Mn, lithology is ultimately an important component in controlling aqueous Mn well water concentrations. Lithologic controls may stem from a variety of geochemical processes. Shales, for example, typically contain higher dissolved organic carbon than other aquifer lithologies, resulting in more anoxic conditions in environments downgradient of shales (Fox et al. 2020).

#### Aqueous Mn and spatial relationships

**Fig. 4:**
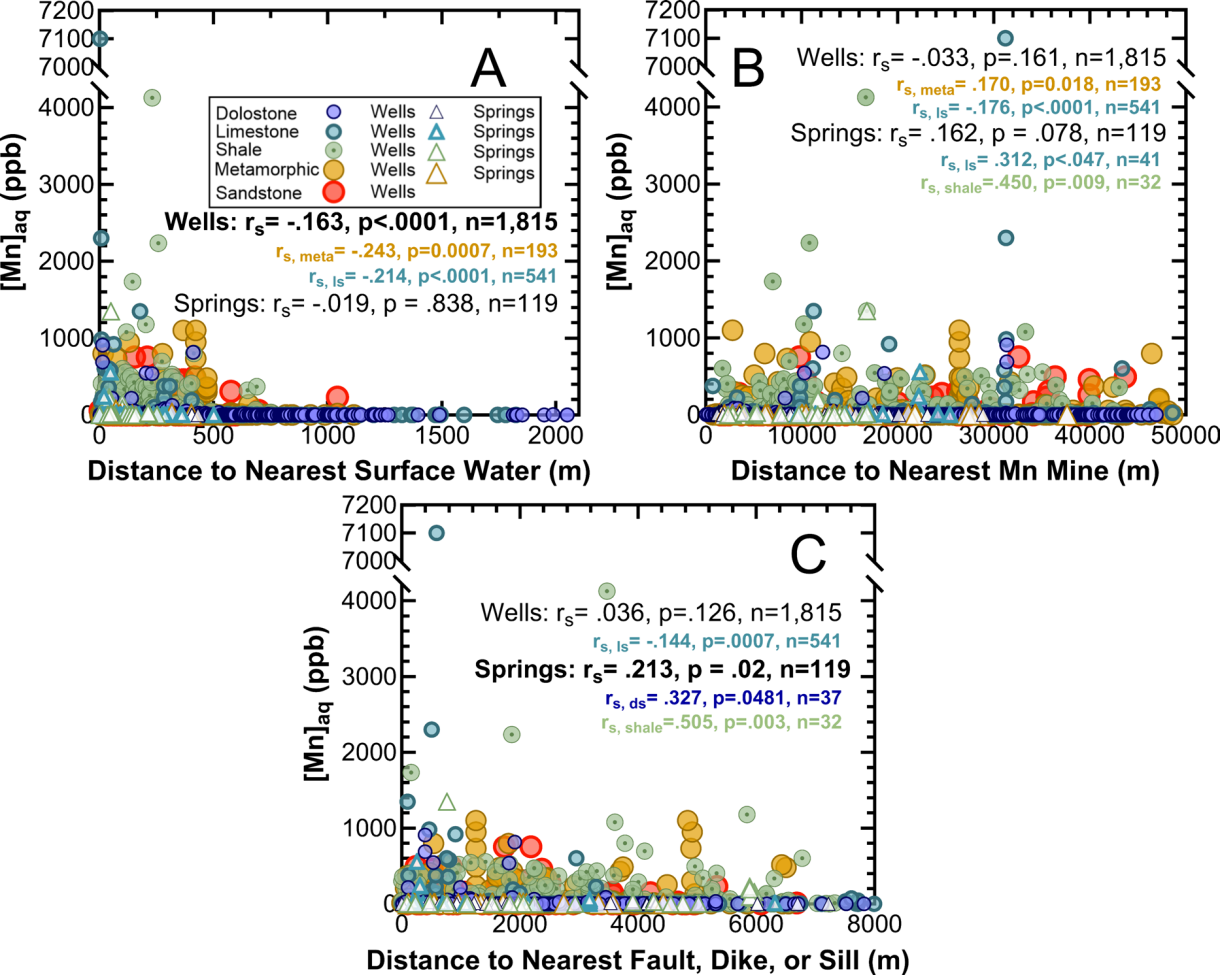
Aqueous Mn concentrations for wells from the VAHWQP data set and the USGS (filled in circles) and for springs from the VAHWQP data set and field sites (open triangles) as a function of distance to nearest surface water feature (**A**), to nearest Mn mine (**B**), and distance to nearest fault or mafic dike or sill (**C**), with those from aquifers primarily comprised of dolostone (dark blue), limestone (teal), shale (green), metamorphic rocks (yellow), and sandstones (red). Nonparametric Spearman correlation coefficients (r_s_; α = 0.05), two-tailed p values (p), and total number (n) reported for the entire well and spring data sets (bolded if statistically significant) as well as for each statistically significant correlation within aquifer lithology subgroups (dolostone denoted as ds, limestone as ls, metamorphic as meta, shale as shale, and sandstone as ss)

The closer a well or spring is to a surface water body in the Shenandoah Valley (Figure S7), the more elevated the aqueous Mn (Fig. 4A). This negative correlation between aqueous Mn concentrations and the distance to the nearest surface water body is only statistically significant for wells as a whole, and for wells sourced from limestone and metamorphic rock aquifers (Fig. 4A). Shallower wells within this data set are typically close to surface water bodies, with a positive weak (yet statistically significant) correlation between well depth and distance to nearest surface water for the wells as a whole, with r_s_ = 0.145, *n* = 1,260, *p* < 0.0001 (Figure S8). By primary aquifer rock type, this positive weak correlation between well depth and surface water is only statistically significant for limestone and metamorphic rocks (as with aqueous Mn and surface water), thus the patterns observed with respect to shallower wells occurring nearer surface water bodies and increasing aqueous Mn nearer surface water bodies are likely interconnected. Interestingly, nearly all deep wells (> 200 m deep) are < 1100 m from a surface water body, and all wells > 1110 m from a surface water body are < 200 m deep (Figure S8). In other words, many shallower wells are far from surface water bodies, and many deep wells are nearby surface water bodies, even if on the whole shallow wells are closer to surface waters (Figure S8). However, wells and springs > 1050 m and > 100 m away from surface water bodies, respectively, are all below 100 ppb Mn (Fig. 4A).

In contrast, well waters and springs with Mn > 100 ppb occur infrequently near Mn ore mines (Figure S9), with just 17 out of the 176 sites within 4000 m of a Mn ore mine exceeding that threshold (Fig. 4B). No statistically significant correlation occurs for aqueous Mn and nearby Mn ore mines for the well and spring data sets as a whole, but examining trends within aquifer lithology reveals that Mn concentrations in wells from metamorphic aquifers and springs from limestone and shale aquifers are positively correlated with distance to Mn ore mines (Fig. 4B). Meanwhile, water wells from limestone aquifers exhibit a very weak negative correlation (Fig. 4B).

With faults often associated with the formation of Mn ores (Carmichael et al., 2017; Kiracofe et al., 2017) as well as with surface water bodies, correlations between aqueous Mn concentrations and distance to nearest fault, dike and sill (Figure S10) is less straightforward. Overall, the well waters most elevated in Mn from limestone aquifers tend to occur closer to these structural features, while springs exhibit a negative correlation between aqueous Mn and distance to faults, dikes, and sills (Fig. 4C). Thus, waters from wells in limestone aquifers consistently show increased aqueous Mn the closer their location is to surface waters, Mn ore mines, and structural features (Fig. 4). However, springs emerging at shale and carbonate lithologies exhibit positive correlations between aqueous Mn and distance to Mn ore mines and these structural features (Fig. 4).

### Mn in soils by soil units and orders

Across all soil orders, spring waters generally exhibited lower average Mn concentrations compared to well waters, with most values falling well below the 100 ppb threshold (Table S3). An exception was noted in the Wallen-Lily-Drypond-Dekalb series, where an average Mn concentration of 228 ppb was recorded in six spring samples, significantly higher than the 100 ppb average observed in wells (Table S3). Conversely, in the Weikert-Berks series, a stark contrast was observed between wells and springs, with an average Mn concentration of only 4 ppb in springs (six samples) versus 109 ppb in wells (273 samples). While these observations are intriguing, the limited sample size precludes definitive conclusions.

**Fig. 5:**
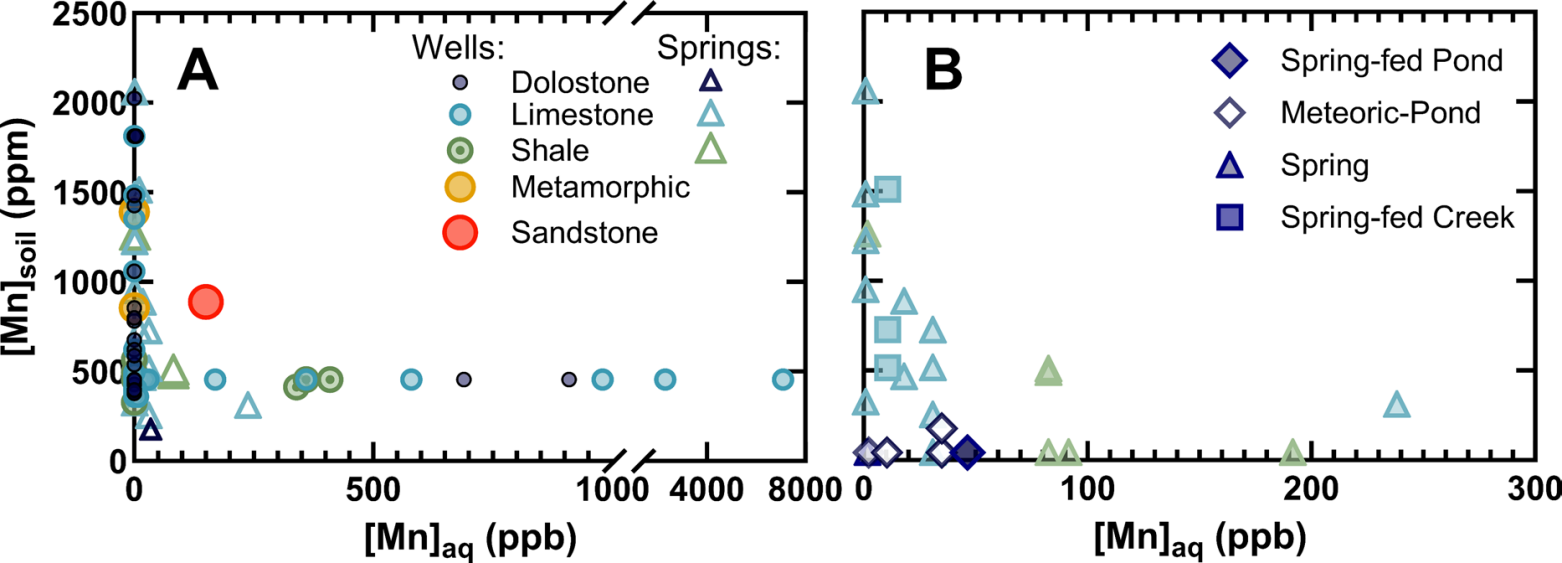
The concentration of solid-associated Mn in soils for each USGS well (circles) and field sampled spring (triangles) in the data set for the Shenandoah Valley, VA as a function of the aqueous Mn concentration in that well and spring (**A**). All field samples (**B**) grouped by sample type, with springs (triangles), spring-fed creeks (squares), spring-fed ponds (filled diamonds), and meteoric ponds (hollow diamonds). Points are color coded by aquifer lithology, with sandstone (red), metamorphic (yellow), shale (green), limestone (teal), and dolostone (dark blue). USGS well water and soil Mn data extracted from McMahon et al., (2018a, 2018b) with soil Mn concentrations originating from Smith et al. (2013)

Examining Mn concentrations in soils and the corresponding aqueous Mn levels in well waters (Smith et al., 2013) and springs (this study) reveals a distinct relationship between the two. Interestingly, soils with Mn concentrations ~ 500 ± 50 ppm appear to be the most associated with heightened levels of aqueous Mn (Fig. 5), regardless of whether that data was obtained via our spring field study or originated from Smith et al. (2013). Wells situated in regions where soils exhibit either notably high or remarkably low Mn concentrations were characterized by minimal aqueous Mn content (Fig. 5A), with only a singular sample from soils > 550 ppm Mn exceeding 100 ppb aqueous Mn in the associated well from a sandstone aquifer (Fig. 5A). A comparable pattern occurs in springs, with springs containing elevated aqueous Mn predominantly occurring in areas where soil Mn concentrations range between 300 and 500 ppm (Fig. 5). However, it should be noted that there are three samples from the field work in this study that contain appreciable concentrations of aqueous Mn but had soil Mn concentrations below detection limits (Fig. 5B). Thus, the field study results are largely consistent with those of wells: elevated aqueous Mn in springs and wells typically occurs with soil Mn ~ 300–550 ppm Mn, but when soil Mn > 1000 ppm, there is a substantial decrease in aqueous Mn values (Fig. 5).

In general, soils classified as alfisols and alfisol-ultisol mixtures consistently exhibit Mn concentrations in wells and springs significantly below the 100 ppb threshold, indicative of lower risk (Table S3). However, considerable variability in Mn levels was observed, as evidenced by the high standard deviation across all soil series (Table S3). Notably, the highest aqueous Mn concentration within all soil orders was recorded in an alfisol, the Frederick-Carbo series (Table S3). Meanwhile, the inceptisol (Weikert-Berks series) and an inceptisol-ultisol mixture (Wallen-Lily-Drypond-Dekalb) demonstrate average aqueous Mn concentrations at or exceeding 100 ppb, conveying an elevated risk (Table S3). These two soil series coincidently run along the Massanutten Mountain region, with aquifers primarily consisting of sandstone and thus it is difficult to deconvolute separate the impact of the Weikert-Berks series and the Wallen-Lily-Drypond-Dekalb series from that of sandstone aquifers (Fig. S2). It should be noted that the one other inceptisol containing unit, the Litz-Groseclose series, only contains two samples, and thus any conclusions drawn regarding that series is of course limited.

### Impact of soil weathering on aqueous Mn isolated by comparing meteoric and spring fed ponds

The Maple Flats Ponds Complex is notable for its limited hydrologic connectivity, with several meteoric ponds and 1 spring-fed pond. This area provides a unique opportunity to study the role of soil weathering on aqueous Mn in springs and other surface waters. These sinkhole ponds form as a result of thick Pleistocene age alluvial deposits (Hack, 1965; Nowroozi et al., 1997) containing appreciable clay fractions overlying sinkhole-ridden karst. These sinkholes form depressions in the landscape that can then fill with water due to the thick impermeable clay cap, with water inputs primarily from precipitation, surface runoff, and interflow (Downey et al., 1999). The soil composition of these closed water bodies [Frog/Oak Pond (FOP), Twin Ponds South and North, and Deep Pond (DP)] may be compared to that of Spring Pond, which is thought to be spring-fed, to determine how great an influence soil weathering has on the redox geochemistry of Mn.

Additionally, the close proximity downgradient and within watershed of the ponds to historic Mn mines [Maple Flats is located near the Kennedy Mn ore mine suite (Figure S11)] allows for the determination of the influence of possible mine drainage and soil weathering on mobilizing Mn mine-associated soil constituents. DP and FOP lie between the Kennedy Mn ore mine region and the rest of the Maple Flats Complex (Figure S11), with DP located ~ 1200 m northeast and 3.2% down gradient of an open Mn ore pit and FOP ~ 1300 m northeast and 4.2% down gradient of one of the Mn ore mine adits.

All soil cores collected from the Maple Flats Ponds Complex were gleyed, with Fe oxidation apparent shortly after collection. Thus, soils were collected and immediately vacuum sealed and placed in a cooler until transferred to a refrigerator at 4 °C. Consistent with previous research finding primarily acidic soils in these sinkhole ponds (Knox, 1997), soil pH was found to be between 3.5 and 5.0. Twin Ponds South and North, DP, and Spring Pond were found to have Mn_soil_ below detection limit (Fig. 5B). Only one soil core from the Maple Flats Complex was found to have Mn_soil_ above XRF detection limit: the core from FOP collected on the near side of the Kennedy Mn Ore Mine with ~ 180 ppm Mn_soil_. Relative to other sites from both carbonate and shale bearing aquifers, these levels of Mn_soil_ are very low (Fig. 5) and thus while mobilization of Mn from historic Mn ore mines may well occur in this region (and therefore be responsible for the soil Mn detected at FOP), such mobilized Mn is not subsequently retained in substantial concentrations in these soils.

All of the sampled waters within these bodies contained aqueous Mn < 100 ppb (Fig. 5B). Classifying the pond waters with the Jurgen et al. (2009) redox classification scheme finds three of the ponds (Spring Pond, FOP, and DP) to be “mixed (oxic-anoxic)” with all others classified as “oxic.” These three ponds also contain the highest aqueous Mn concentrations (10.5 ppb–46.4 ppb Mn) in the sinkhole pond system, as well as appreciable aqueous Fe concentrations (7–45 ppm Fe). The aqueous Mn concentrations for Spring Pond, FOP, and DP (the three classified as “mixed (oxic-anoxic)”) are roughly between the 75–90th percentiles for both water wells and springs (Table 1). Of particular note regarding these three ponds is that FOP and DP are the two ponds that lie between the relict Kennedy Mn Ore mine and the rest of the sinkhole pond complex, and that Spring Pond is the only pond within the Maple Flats Complex with likely groundwater inputs (Buhlmann et al., 1999). The waters of Spring Pond contain the greatest aqueous Mn concentrations in the Maple Flats Complex (40.0–46.4 ppb). These results therefore suggest that groundwater baseflow can further increase aqueous Mn, and that soil-derived Mn can contribute appreciable amounts of aqueous Mn in this region (either originating from Mn ore mines or authigenic Mn) if the resulting waters maintain somewhat reducing conditions.

### Soil Mn redox state relationships

**Fig. 6:**
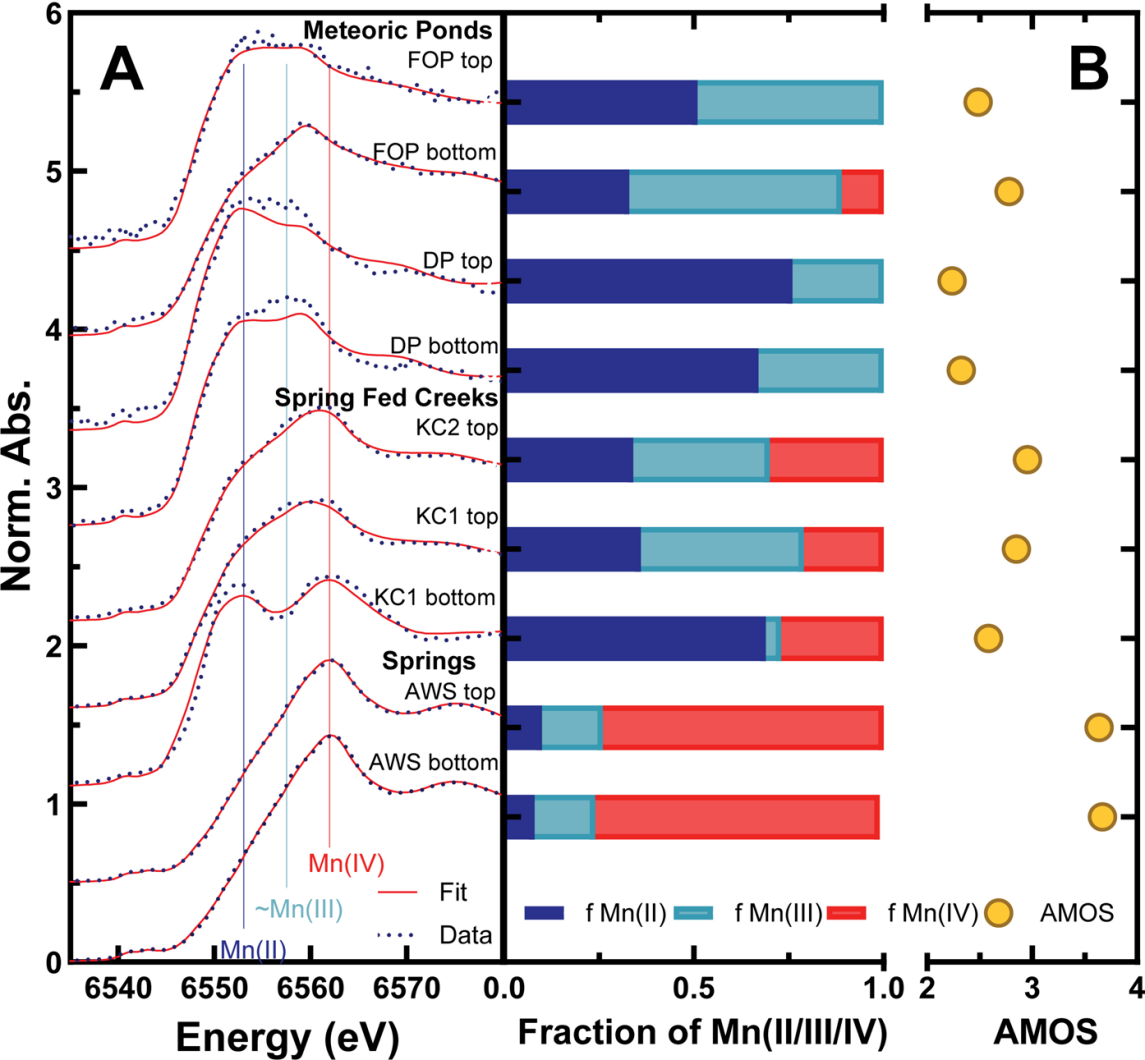
XANES spectral data (black dotted lines) plotted against LCFs (red straight lines) (**A**) used to calculate the corresponding average Mn oxidation state (AMOS; yellow circles) and fractions of solid-associated Mn(II) (dark blue bars), Mn(III) (teal bars), and Mn(IV) (red bars) (**B**). Vertical straight lines at 6553 eV, 6558 eV, and 6562 eV correspond to Mn(II), ~ Mn(III), and Mn(IV)

The XANES spectral LCFs of soil samples reveal differences in soil Mn oxidation states in different site types (Fig. 6A). The meteoric pond samples analyzed from the Maple Flats Pond Complex predominantly indicate the presence of reduced Mn, mainly Mn(II) and Mn(III), with an exception in the FOP bottom sample which was found to contain ~ 11% Mn(IV). Notably, the DP top and DP bottom samples demonstrate a substantial Mn(III) component, as evidenced by the prominent features corresponding to Mn(III) in the Mn K-edge XANES spectra at ~ 6558 eV (Fig. 6A). However, the offset between the LCF and sample XANES spectra for these two samples at ~ 6558 eV (Fig. 6A) indicates that the LCFs do not adequately capture the Mn(III) component in these two samples, suggesting a likely underrepresentation of Mn(III) in the fractions of Mn(II/III/IV) and average Mn oxidation state determinations made via LCFs for these two samples (Fig. 6B).

In contrast, soil samples adjacent to spring or spring-fed creeks contain appreciable amounts of Mn(IV) (all > 20%) (Fig. 6B). In spring-fed creek samples, the two samples from the uppermost portion of soil cores reveal nearly equal proportions of Mn(II) (Fig. 6B). Conversely, the KC1 bottom sample was characterized by only a minor presence of Mn(III), with Mn(II) being the most abundant, followed by Mn(IV). Meanwhile, the top and bottom of the soil core analyzed by XANES deriving from a spring site (ASW) are very similar to one another with respect to Mn oxidation, with substantial fractions of Mn(IV) (~ 75%), followed by Mn(III) at 16% and Mn(II) at ~ 9% (Fig. 6B).

**Fig. 7:**
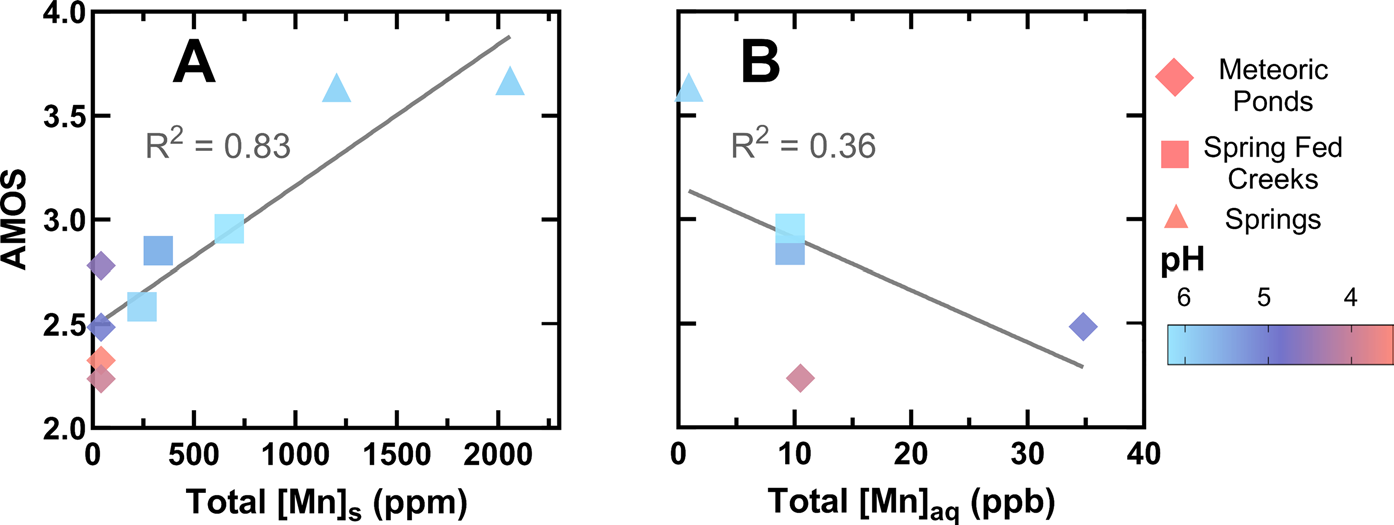
XANES spectroscopy-derived average Mn oxidation state (AMOS) of all soils analyzed by XANES as a function of the total Mn concentration in soils (**A**) and all top fractions of soils as a function of the aqueous Mn concentration in nearby water features (**B**). Water feature types are denoted by symbols, with meteoric ponds (diamonds), spring fed creeks (squares), and springs (triangles), and the symbol colors correspond to soil pH as distributed by a gradient scale (pH < 4 as pink, 4–5 as purple, > 5 as blue). Lines of best fit are included for the two data sets as well as corresponding R^2^ values

A significant positive correlation was observed between the AMOS and total soil Mn concentration, with a determination coefficient (R^2^) of 0.83 (Fig. 7A). This correlation indicates that higher soil Mn concentrations correspond to increased AMOS, while lower soil Mn concentrations are associated with decreased AMOS. Additionally, soils exhibiting lower AMOS were found to have more acidic soil pH (Fig. 7A). With more acidic conditions promoting reduced Mn(II) over that of Mn(III) and Mn(IV), these results are consistent with predicted Mn redox behavior in natural systems (Luther III, 2005).

In the quantification of soil Mn, each soil sample analyzed via XANES was paired with a corresponding soil Mn concentration, determined using XRF. In contrast, for the comparison between XANES-derived soil AMOS and aqueous Mn levels, a single aqueous Mn concentration was assigned to each sampling site, as these measurements were referenced to a common water source, such as a spring or a pond. This comparison necessitates a choice between available samples, namely the “bottom” or “top” suite of samples; overall the results are consistent between using either the bottom or the top suite of samples. However, given the limited availability (i.e., four bottom samples and five top samples), a decision was made to proceed with the analysis of top soil samples, as that grouping contains more samples. A moderate negative correlation (R^2^ = 0.36) was identified between the aqueous Mn concentrations in nearby water features and the AMOS of the associated soil Mn in the top soil fractions (Fig. 7B). Due to limitations in the overall number of spring-associated soil samples able to be analyzed by XANES spectroscopy in this study, inferences regarding site types cannot be made for this data set. However, these relationships between soil AMOS, soil Mn, and aqueous Mn of nearby water features (Fig. 7) do suggest that higher soil Mn and AMOS correspond to lower aqueous Mn concentrations, consistent with the XRF results obtained for a much larger number of samples as shown in Fig. 5.

**Fig. 8:**
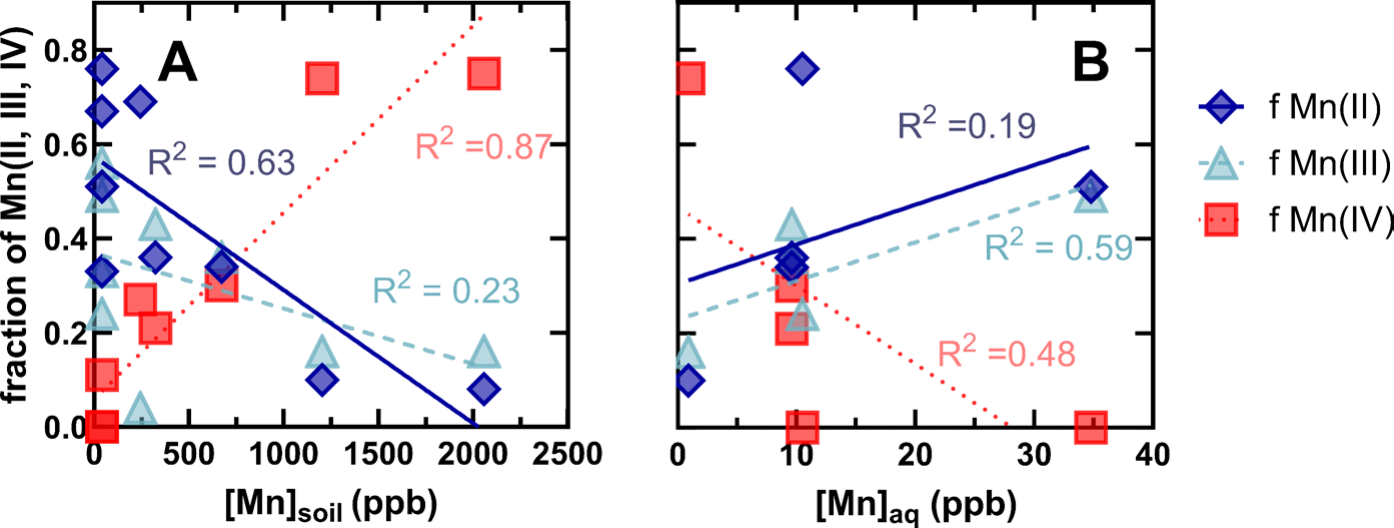
XANES spectroscopy-derived fractions of Mn(II) (red squares), Mn(III) (teal triangles), and Mn(IV) (blue diamonds) of all soils analyzed by XANES as a function of the total Mn concentration in soils (**A**) and all top fractions of soils as a function of the aqueous Mn concentration in nearby water features (**B**). Corresponding lines of best fit and R^2^ values for Mn(II) (red dotted line), Mn(III) (teal dashed line), and Mn(IV) (blue solid line) are included

The AMOS LCF results also reveal correlations between individual Mn redox state fractions [Mn(IV), Mn(III), and Mn(II)] and soil Mn concentrations. Mn(IV) shows a strong positive correlation with soil Mn (R^2^ = 0.87), while Mn(II) exhibits a negative correlation (R^2^ = 0.63) (Fig. 8A). These trends align with the overall positive relationship between soil Mn concentrations and AMOS, as higher Mn(IV) and lower Mn(II) fractions contribute to increased AMOS. Mn(III) displays a weak negative correlation with soil Mn concentrations (R^2^ = 0.63) (Fig. 8A). However, considering the substantial underestimation of Mn(III) in the DP top and bottom samples, adjusting for these disparities could result in a stronger negative correlation between Mn(III) and total soil Mn content. Examining the fractions of Mn(IV), Mn(III), and Mn(II) as a function of aqueous Mn concentrations of the nearby water features reveals, as expected based on AMOS vs. [Mn]_aq_, the opposite trends. For example, soil Mn(II) and Mn(III) are positively correlated with aqueous Mn(II) (Fig. 8B). Non-parametric correlations find that the only statistically significant correlations between aqueous Mn and all other soil parameters measured for this subset of soil samples are the AMOS, [Mn(II)]_soil_, fraction of Mn(III), fraction of Mn(IV), [Mn(IV)]_soil_, total [Mn]_soil_, and soil pH (Figure S12). While there are clear limitations due to the relatively low number of samples with associated XANES spectra, the results are consistent.

## Discussion

### Relative risk of Mn exposure in Shenandoah Valley

This study highlights a differential risk profile between springs and water wells in terms of Mn contamination. Springs are observed to confer a lower risk compared to water wells, with waters originating in dolostone aquifers presenting the lowest risk for Mn contamination than other aquifer types. Springs likely represent redox transition zones, with both young (0–8 year old) water and old water present in karst springs in the Shenandoah Valley (Yager et al., 2013). Despite limestone aquifers showing low average Mn concentrations, a substantial number of water wells and springs within these limestone formations exhibit high Mn levels, often associated with shallower wells in close proximity to surface waters, Mn ore mines, and structural features. This inconsistency necessitates routine monitoring of Mn contamination in drinking water sources sourced from limestone, particularly in limestone-sourced wells near surface water features, Mn ore mines, and structural features such as faults, dikes, and sills. Conversely, shale formations are identified as having the highest average Mn concentrations and also yield the most concentrated samples in both water wells and springs. These results underscore the need for regular assessment of Mn contamination in drinking water sources from shale aquifers. Sandstone aquifers, while not reaching the peak Mn levels observed in shales and limestones, demonstrate on average higher Mn concentrations than other lithologies. Thus, it is advisable to assess sandstone aquifer-derived water for Mn contamination before its use as drinking water in the Shenandoah Valley, VA.

This research also reveals a correlation between the proximity to surface water (SW) and Mn contamination risk, with sources farther from SW presenting a lower risk than those closer, consistent with previous findings assessing USGS wells across the US (McMahon et al., 2018a). Additionally, deeper wells exhibit lower Mn contamination risk compared to more shallow wells. This finding contradicts the initial assumption that deeper wells, being more reducing, would have higher Mn concentrations. The observed higher Mn in shallower wells likely results from their proximity to surface water, where the water table is generally shallower, necessitating less deep well drilling. It is possible that the interconnected nature between well depth, proximity to surface water, and Mn concentration stems from the higher dissolved organic carbon concentrations previously observed for groundwater wells near surface waters, which would promote reducing conditions and therefore Mn mobilization (McMahon et al., 2018a).

Soil types in the Shenandoah Valley also likely play a substantial role in Mn contamination risk. Springs and wells in soils dominated by alfisols generally show lower Mn concentrations, suggesting a lower risk profile. In contrast, inceptisols and their mixtures exhibit higher Mn levels. The variability and exceptions observed in specific soil series highlight the complexity of Mn distribution in soils in this region, necessitating further research with larger sample sizes for a more comprehensive understanding of these dynamics.

### Fe as an indicator of relative Mn risk in the Shenandoah Valley

The interplay between Fe and Mn in the Shenandoah Valley provides crucial insights into the environmental conditions influencing Mn mobilization. The correlation between aqueous Fe and Mn concentrations suggests that redox conditions, rather than factors like pH or alkalinity, are predominant in controlling Mn mobilization in this region.

The Shenandoah Valley's geological backdrop, characterized by ancient, well-weathered carbonates, forms a karst terrain that inherently creates more oxidizing conditions relative to other aquifer types. In aquifers where Fe concentrations are below the indicative threshold of 100 ppb–such as those typical in dolostone and limestone–the conditions are likely more oxidizing. This oxidation state is favorable for the formation of Mn(III/IV) (oxyhydr)oxides, contributing to lower soluble Mn levels in these carbonate aquifers. This finding is aligned with the observed lower risk of Mn contamination in dolostone aquifers, as discussed earlier in the results (Sect. 3.1.1), although reducing conditions (as inferred by aqueous [Fe]) do occur locally in some carbonate wells, coincident with higher Mn concentrations. There are some instances of well waters from dolostone aquifers containing high aqueous Fe (> 2 ppm) yet with low aqueous Mn (as discussed in Sect. "Aqueous Mn and depth profiles"). These instances of high aqueous Fe with low aqueous Mn wells in carbonate aquifers could potentially result either from low initial Mn within the aquifer or due to carbonate stabilizing Mn in the solid phase through the precipitation of Mn-carbonate minerals (e.g., rhodochrosite). With anoxic groundwater in carbonate aquifers often supersaturated with respect to both the Fe and Mn-carbonate carbonate minerals siderite and rhodochrosite (Amirbahman et al., 1998; Bjerg et al., 1995; Jensen et al., 1998), differences in solubility products and precipitation kinetics (Duckworth & Martin, 2004; Jensen et al., 2002) could explain systems that contain high aqueous Fe but low aqueous Mn due to rhodochrosite precipitation.

The data reveal a distinct contrast in Mn risk between carbonate and non-carbonate aquifers. For instance, shale and sandstone aquifers, typically associated with higher Fe concentrations, indicate more anoxic conditions and consequently greater Mn mobilization. This relationship is evident from the higher average Mn and Fe concentrations in well waters and springs sourced from these lithologies, as previously outlined in Fig. 2 and Table S3. The relationship between Fe and Mn thus serves as a critical determinant of the relative risk of Mn contamination in different aquifer types within the Shenandoah Valley. Meanwhile, wells in limestone aquifers tend to have lower aqueous Mn and Fe, yet exhibit increasing Mn when these wells are shallow or close to surface waters, Mn ore mines, or structural features.

Redox controls are likely involved in the association between surface waters and structural features with aqueous Mn in limestone aquifers. The spatial distribution and depth profile analyses (see Sects. 3.1.2 and 3.1.3) support the notion that closer proximity to surface waters and shallower well depths correspond to higher Mn levels. These results are consistent with those of McMahon et al. (2018a), which found increased Mn with decreased distance to surface waters, and with Gillespie et al. (2016), which found that aqueous Mn concentrations in groundwater reach a maximum just below the water table. Yager et al. (2013) suggests faults serve as conduits for old groundwater in the Shenandoah Valley, resulting in nearly vertical upwelling of old groundwater to the surface, and thus nearby shallow water wells could include a component of older, potentially more anoxic, groundwater. In both these scenarios, these conditions likely enhance the dissolution of Mn(III/IV) oxides and the subsequent mobilization of Mn(II), contributing to elevated Mn concentrations in groundwater and spring water sources.

Thus, the observed correlations between Fe and Mn across various lithologies and water sources in the Shenandoah Valley point to redox conditions as a primary control on Mn mobilization. This understanding is crucial for predicting and managing the risk of Mn contamination in groundwater and spring water, particularly in karst terrains like the Shenandoah Valley. The role of Fe as an indicator of redox conditions offers a valuable tool for assessing the relative risk of Mn exposure, guiding both scientific investigation and public health policy. Aqueous Fe(II) rapidly undergoes oxidative precipitation to Fe(III) (oxyhydr)oxide minerals such as hematite, goethite, or ferrihydrite, upon exposure to oxic environments (Millero, 1989) such as exiting household taps, while Mn(II) oxidation kinetics are much more limited (Diem & Stumm, 1984; Morgan, 2005). It is therefore advisable that any household experiencing red or orange staining from drinking water test their water for elevated Mn as well.

### Role of soils in spring and well water Mn in the Shenandoah Valley, VA

In the Shenandoah Valley, the interplay between soil composition and manganese (Mn) concentrations in spring and well water is an important aspect of the regional hydrogeochemistry. With previous research finding considerable uptake of soil Mn by plants as Mn(II), followed by rapid oxidation to Mn(III) during plant decomposition (Herndon et al., 2014), it should be noted that seasonality effects may well be at play with respect to soil Mn-aqueous Mn interactions. A limitation of the current study is that these potential seasonal effects are not explored, due to the fact that the field season was limited to the summer months and sample collection for the VAHWQP data set occurs from February through November. The Valley's soil profile, characterized predominantly by alfisols, ultisols, and inceptisols, presents a complex matrix that may potentially influence Mn dynamics.

Of the three main soil orders prevalent in the Valley (alfisols, ultisols, and inceptisols), alfisols and ultisols are rich in clay and contain significant quantities of iron and aluminum oxides. Ultisols, being more weathered, have higher iron oxide contents and tend to be more acidic (Soil Survey Staff, 1999). This weathering potentially contributes to the mobilization and bioavailability of Mn in these soils, as indicated by the relatively lower Mn concentrations in waters interacting with alfisols compared to ultisols.

Representing the least developed soils in the region, inceptisols have low organic carbon content. The current study observed that inceptisols and their mixtures often present higher levels of Mn in well and spring waters. This trend suggests a distinct role of these soils in Mn mobilization, potentially due to their undeveloped nature allowing easier leaching and transfer of Mn into water sources. However, as soil is ultimately a product of the parent material, it is difficult to separate the extent of soil and lithologic controls in this region.

However, the observed correlations between soil Mn redox states, soil Mn concentrations, and aqueous Mn concentrations are intriguing in that they suggest soil–water interactions are present. In particular, increased soil Mn(III) content with elevated aqueous Mn(II) concentrations may be indicative of aqueous Mn(II)-solid Mn(IV) comproportionation reactions [which generate 2Mn(III)] occurring in the soil matrix, thereby leading to the formation of elevated solid Mn(III). These comproportionation reactions, occurring under specific redox conditions with elevated aqueous Mn(II), are indicative of the oxic-anoxic interface. In addition, the soil and water geochemistry of the Maple Flats Complex indicates a potential role of soil–water interactions leading to elevated Mn due to redox conditions. With Spring Pond, FOP, and DP all classified as “mixed (oxic-anoxic)” while the other ponds in the Maple Flats Ponds Complex are classified as “oxic” (via the Jurgen et al. (2009) redox classification scheme), it is quite possible the redox state of these “mixed (oxic-anoxic)” ponds with slightly elevated Mn is resulting in stabilization of Mn(II) in the aqueous phase, whether that aqueous Mn(II) is the result of soil weathering or groundwater inputs.

### Contextualizing Mn contamination in the Shenandoah Valley: comparison with prior studies

Our research has identified the prevailing redox state of aquifers and soils as the most likely controls on Mn mobilization and risk in springs and water wells in the Shenandoah Valley. With Mn commonly precipitating out as Mn hydroxides in karst and thus are transported largely as particulates (along with other associated metal contaminants) (Vesper et al., 2003), the aquifer redox state may in turn exert a broader control on contaminant transport in the Shenandoah Valley region. Consistent with findings by Gillispie et al. (2016) and McMahon et al. (2018a), our study confirms the strong link between soil geochemistry, soil redox parameters, and Mn concentrations in water wells and springs. We extend this understanding by demonstrating the role of soil types and distribution of soil-associated Mn redox states in Mn risk dynamics within the Shenandoah Valley.

Like the McMahon et al. (2018a) assessment of wells across the United States, we find that proximity to nearby surface waters is associated with elevated Mn in associated water wells, and that this association extends to springs as well. These results are also in agreement with those beyond the United States, finding reducing floodplains (de Meyer et al., 2017, 2023) and aquifers to confer higher Mn risk (Kousa et al., 2021). While initially our findings that aqueous Mn decreases with well depth seems to be at odds with the concept that reducing environments lead to more Mn risk, it is consistent with the Gillespie et al. (2016) study on wells in the Piedmont region of North Carolina which found the maximum aqueous Mn levels are present just below the water table as a result of chemical weathering of saprolite. While the geology of these two regions are distinct, the similarity in results indicates that chemical weathering and soil dynamics play an important role in overall extent of Mn risk in the Shenandoah Valley.

Our research has pinpointed drinking water sources originating in shale and sandstone aquifers as particularly susceptible to elevated aqueous Mn, with 21.2% and 26.8% of wells yielding aqueous Mn in excess of 100 ppb in shale and sandstone aquifers, respectively (Table 1). Meanwhile ancient, weathered karst terrains confer a protective effect, with just 1% and 2.6% of wells in dolostone and limestone aquifers exceeding 100 ppb aqueous Mn (Table 1). Similarly, Gillespie et al. (2016) identified differences in rock types and well water Mn risk in the Piedmont region of North Carolina, but with a metamorphic zone (the Carolina Slate Belt) as containing the highest Mn, with an average of 110 ± 210 ppb Mn. Another study on Mn in well waters, also focused on the Piedmont region but in Virginia’s Roanoke River Watershed (to the southeast of our region of interest) also found that metamorphic rocks are associated with higher Mn risk, finding that marbles (yielding high aqueous Ca) were associated with higher aqueous Mn (Kiracofe et al., 2017). Meanwhile, our results found that metamorphic rocks were, on average, below thresholds of concern with respect to Mn, yielding aqueous Mn values on average of 50 ± 160 ppb. It should be noted that the metamorphic rocks within the Shenandoah Valley (including quartzites, greenstones, and gneisses) are distinct from those in the Piedmont region, which includes more marbles, slates, and schists. In higher latitudes, glacial sediments containing reducing groundwater increases Mn risk in drinking water (Erickson et al., 2019). Thus, the differences in results suggest that lithology is an important factor with respect to aqueous Mn in associated well waters and springs, particularly in regions like the Shenandoah Valley in which surface lithology is largely representative of primary aquifer rock types. However, the exact lithology that poses the most risk with respect to Mn likely varies from region to region.

## Conclusion

The Shenandoah Valley provides a unique backdrop for investigating the mobilization of Mn in spring and well water systems in karst terrain due to the presence of both karst and non-karst aquifers. Given the reliance of much of the global population on well water and springs originating from karst aquifers, understanding the main drivers of Mn contamination in karst is critical.

Our findings suggest that redox conditions are the primary driver influencing aqueous Mn concentrations in Shenandoah Valley's water sources. The data indicates that karst terrains yield more oxidizing groundwaters, thereby limiting Mn solubility. Conversely, reducing conditions (prevalent in shale and sandstone aquifers and near surface waters) facilitate more Mn mobilization to solution. Thus, regular monitoring of Mn levels in drinking water sourced from groundwater, especially in areas underlain by anoxic aquifers or in proximity to surface waters, is essential.

The variability in Mn concentrations across different soil orders and lithologies underscore the complexity of Mn dynamics in the Shenandoah Valley. Thus Mn risk assessment in the region should consider both soil composition and factors that can influence both the soil and aquifer redox state. Understanding these relationships is essential for developing effective management strategies to mitigate Mn-related health risks and ensure the safety of water resources in the Shenandoah Valley and other karstified regions.

## Competing interests

The authors have no relevant financial or non-financial interests to disclose.

### Supplementary Information

Below is the link to the electronic supplementary material.

Supplementary file1 (DOCX 5571 KB)

Supplementary file2 (XLSX 25 KB)

## Data Availability

Soil and water geochemistry data from the field study portion of this research can be downloaded in the Supplementary Information Spreadsheet. Data from VAHWQP are not available due to privacy agreements with study participants. More information can be found at www.wellwater.bse.vt.edu. Data from the USGS used in this study are available at https://doi.org/10.5066/P9Y4GOFQ.
