# Supplementary material for: Manganese exposure from spring and well waters in the Shenandoah Valley: interplay of aquifer lithology, soil composition, and redox conditions
Source: Environ Geochem Health. 2024 May 2;46(6):203. doi: 10.1007/s10653-024-01987-4 (PMC11065944; doi:10.1007/s10653-024-01987-4)
Supplement: Supplementary file 1 — Supplementary file1 (DOCX 5571 KB) [file 10653_2024_1987_MOESM1_ESM.docx]

**Supplementary Information for:**

**Mn exposure from spring and well waters in the Shenandoah Valley: Interplay of aquifer lithology, soil composition, and redox conditions**

Margaret A. G. Hinkle*^a^, Brady Ziegler^b^, Haley Culbertson^a^, Christopher Goldmann^b^, Marina A. Croy^a^, Noah Willis ^c^, Erin Ling ^d^, Benjamin Reinhart ^e^, Eva C. Lyon^a,f^

*Corresponding author: Tel.: +1-540-458-8271; E-mail: hinklem@wlu.edu

^a^Department of Earth and Environmental Geoscience, Washington & Lee University, 204 W. Washington Street, Lexington, VA 24450, USA

^b^Department of Geosciences, Trinity University, 1 Trinity Pl, San Antonio, TX 78212, USA

^c^Geology Department, Whitman College, 345 Boyer Ave, Walla Walla, WA 99362, USA

^d^Department of Biological Systems Engineering, Virginia Tech, Blacksburg, VA 24061, USA

^e^Argonne National Laboratory, Chicago, Illinois 60439, USA

^f^Now at: Department of Geological Sciences, Ohio University, Athens, OH 45701, USA

This Supplementary Information contains 13 pages, 3 tables, and 12 figures

**Table of Contents:**

| Figure S1. Map of springs and wells overlying lithologic map………………….. | ....p. S1 |
| --- | --- |
| Figure S2. Map of springs and wells overlying soil map………………………… | ....p. S2 |
| Figure S3. Mn oxidation state standards XANES spectra………………………. | ....p. S2 |
| Figure S4. Aqueous [Mn] vs. [DO], [DOC], pH, and Hardness…………………. | ....p. S3 |
| Figure S5. Aqueous [Mn] vs. [NO3], [Fe], [Mg], [Ca], [Si], and [SO4]…………. | ....p. S4 |
| Figure S6. Well Depth vs. [Mn], [Fe], [Si], and [Mg]……………………………… | ....p. S5 |
| Figure S7. Map of springs and wells overlying surface water map……………. | ....p. S6 |
| Figure S8. Well Depth vs. Distance to Surface Water and nitrate……………… | ....p. S7 |
| Figure S9. Map of springs and wells with Mn ore mine locations………………. | ....p. S8 |
| Figure S10. Map of springs and wells with geologic structural features………. | ....p. S9 |
| Figure S11. Map of Maple Flats Complex and nearby Kennedy Mn Ore Mine... | ..p. S10 |
| Figure S12. Spearman’s Rho correlation matrix for 9 soil samples…………… | ..p. S11 |
| Table S1. Nonparametric test statistics for [Mn]_aq_ and aquifer rock……………. | ..p. S12 |
| Table S2. Aqueous Fe concentrations by water source type and lithology……. | ..p. S12 |
| Table S3. Aqueous Mn concentrations by soil map unit………………………… | ..p. S13 |

**
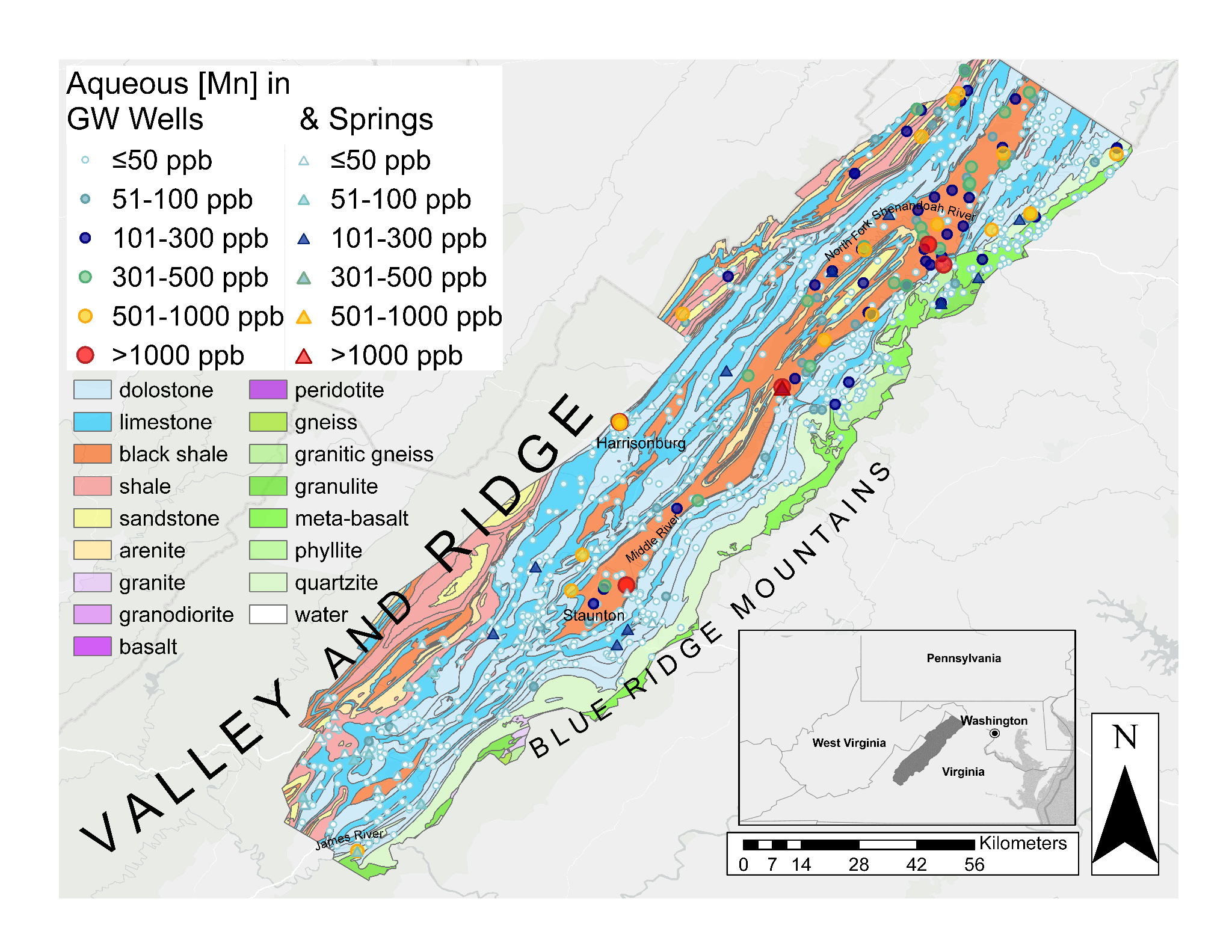
**

**Figure S1.** Spatial distribution of sampling sites for springs (triangles) and water wells (circles) from private drinking water sources from the VAHWQP data set and the USGS groundwater well network overlying the surface lithology of the region, with carbonates (blue), shales (red), sandstones and arenites (yellows), igneous rock (purples), and metamorphic rocks (greens). Sites are color coded by [Mn]_aq_, with values below the threshold of concern for chronic aqueous Mn exposure of 100 ppb as blue, those between 101-300 ppb (yellow), 301-500 ppb (orange), 501-1000 ppb (red), and >1000 ppb (dark red).

**
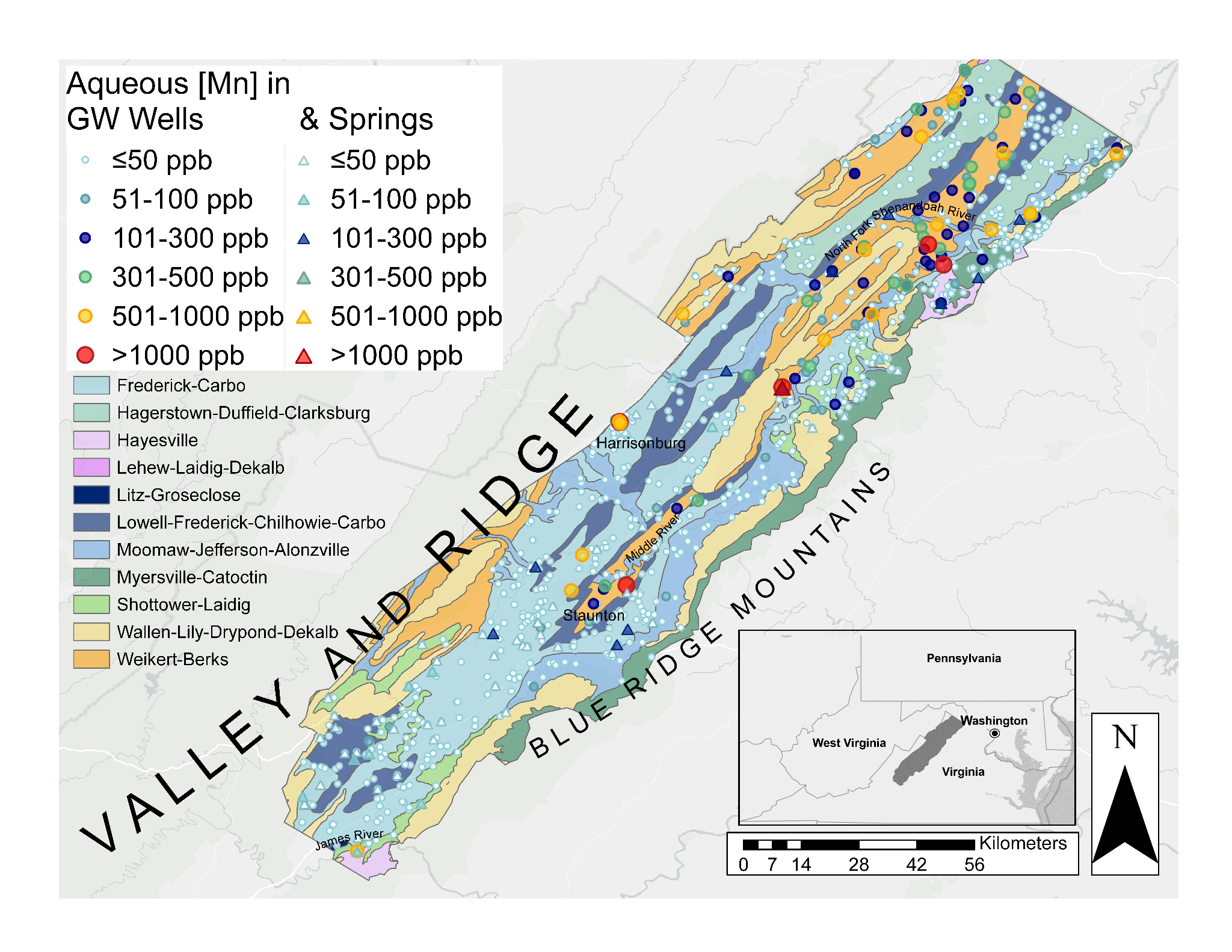
**

**Figure S2.** Spatial distribution of sampling sites for springs (triangles) and water wells (circles) from private drinking water sources from the VAHWQP data set and the USGS groundwater well network overlying the primary soil types from the SSURGO database. Sites are color coded by [Mn]_aq_, with values below the threshold of concern for chronic aqueous Mn exposure of 100 ppb as blue, those between 101-300 ppb (yellow), 301-500 ppb (orange), 501-1000 ppb (red), and >1000 ppb (dark red).


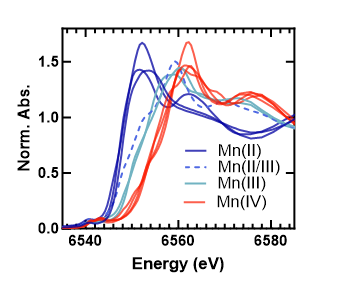


**Figure S3.** Mn K-edge XANES spectra of average Mn oxidation state standards used for the linear combination fits of XANES spectra of soil samples, with Mn(IV) standards in red [pyrolusite, ramsdellite, KBi (a K^+^-birnessite), and Ca_2_Mn_3_O_8_ from Manceau et al. (2012)], Mn(III) standards in light teal (manganite, feitknechtite, bixbyite), Mn(II/III) standard in dashed blue (hausmannite), and Mn(II) standards in dark blue (rhodochrosite, manganese chloride, manganese sulfate).

**
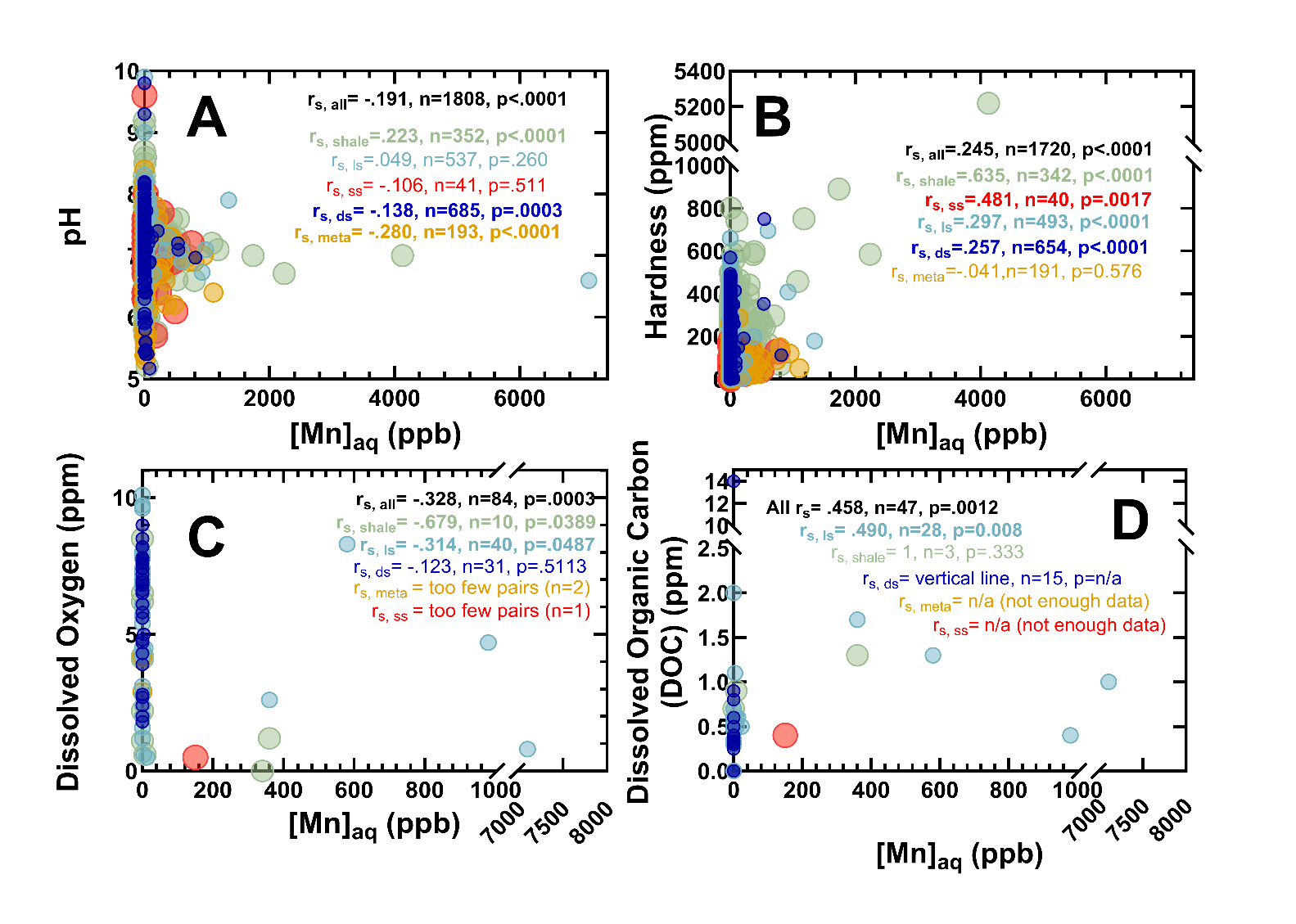
**

**Figure S4.** Aqueous Mn concentrations for parameters commonly associated with Mn solubility and/or indicative of the overall redox state of groundwater: pH (A), hardness (B), dissolved oxygen (C), and dissolved organic carbon (C). Symbols are color coded by aquifer primary rock types with dolostone (dark blue), limestone (bright blue), shale (green), metamorphic (yellow) and sandstone (red).Nonparametric Spearman correlation coefficients (r_s_; α = 0.05), total number (n), and two-tailed p values reported for the entire data set (r_s, all_) as well as for each aquifer lithology subgroup (dolostone denoted as ds, limestone as ls, metamorphic as meta, shale as shale, and sandstone as ss), with statistically significant correlations bolded.

**Figure S5.** Aqueous Mn concentrations versus other major aqueous elements and ions associated with Mn solubility, with redox state, or with different aquifer lithologies: nitrate (A), iron (B), magnesium (C), calcium (D), silicon (E), and sulfate (F). Symbols are color coded by
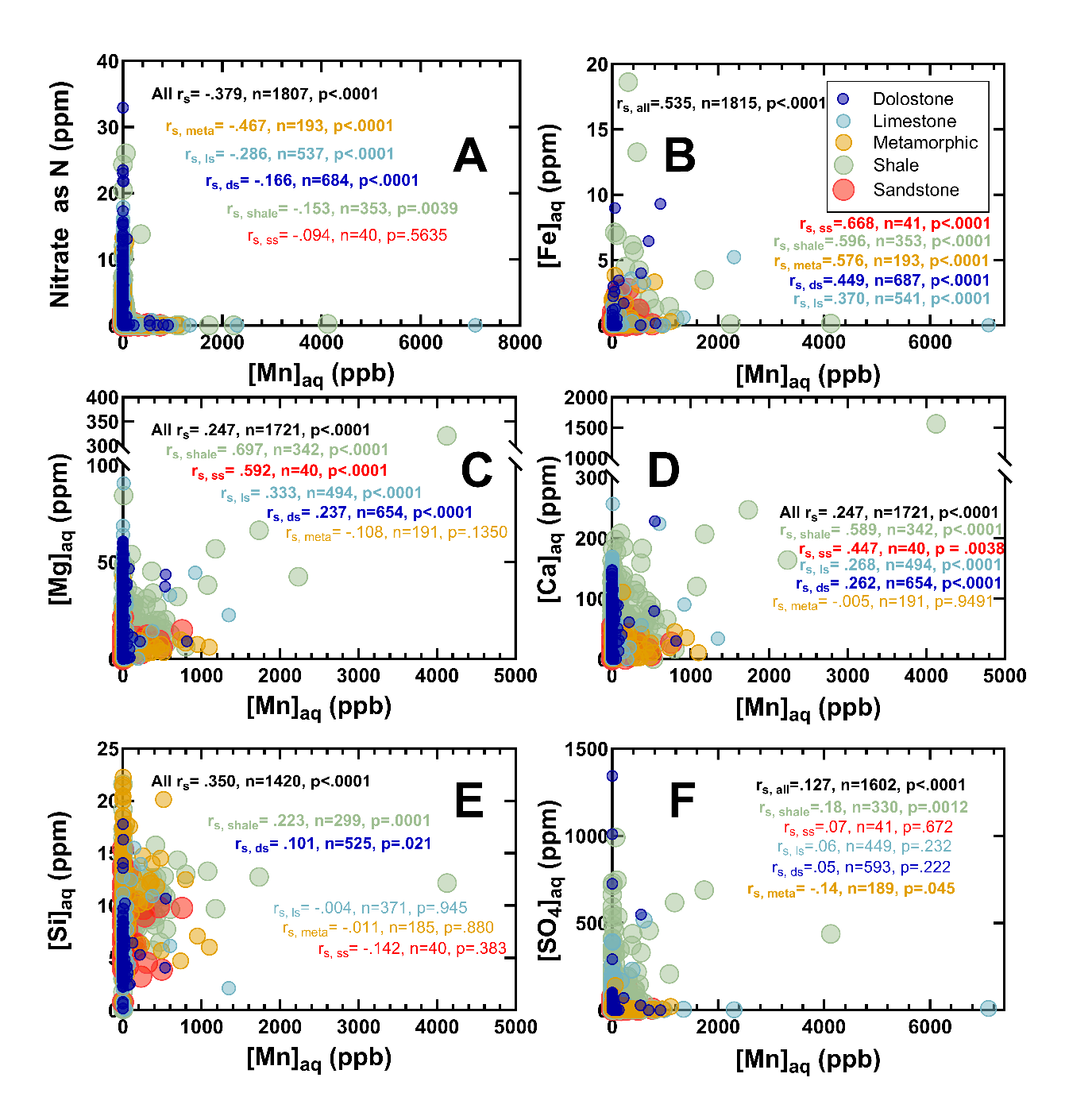
aquifer primary rock types with dolostone (dark blue), limestone (bright blue), shale (green), metamorphic (yellow) and sandstone (red). Nonparametric Spearman correlation coefficients (r_s_; α = 0.05), total number (n), and two-tailed p values reported for the entire data set (r_s, all_) as well as for each aquifer lithology subgroup (dolostone denoted as ds, limestone as ls, metamorphic as meta, shale as shale, and sandstone as ss), with statistically significant correlations bolded.

**
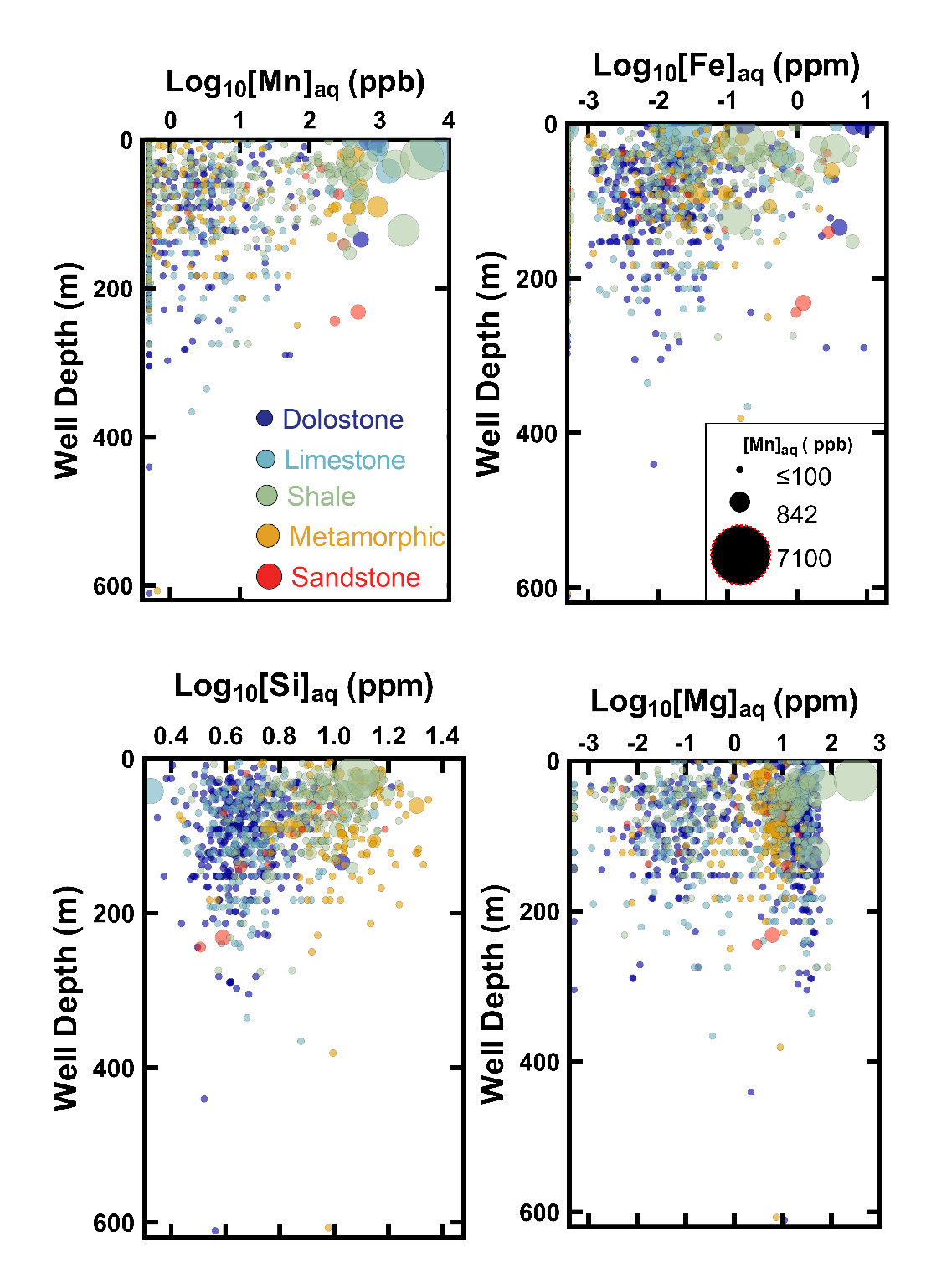
**

**Figure S6.** Well Depth vs aqueous concentrations of Mn, Fe, Si, and Mg, with wells in aquifers with primary lithology comprised of dolostone (smallest dark blue circles), limestone (light blue), shale (green), metamorphic (yellow), and sandstone (largest red circles). Note: the same number of samples are plotted in each panel; many data points for Mn and Fe are near 0 and thus do not appear to be as populated as the others.


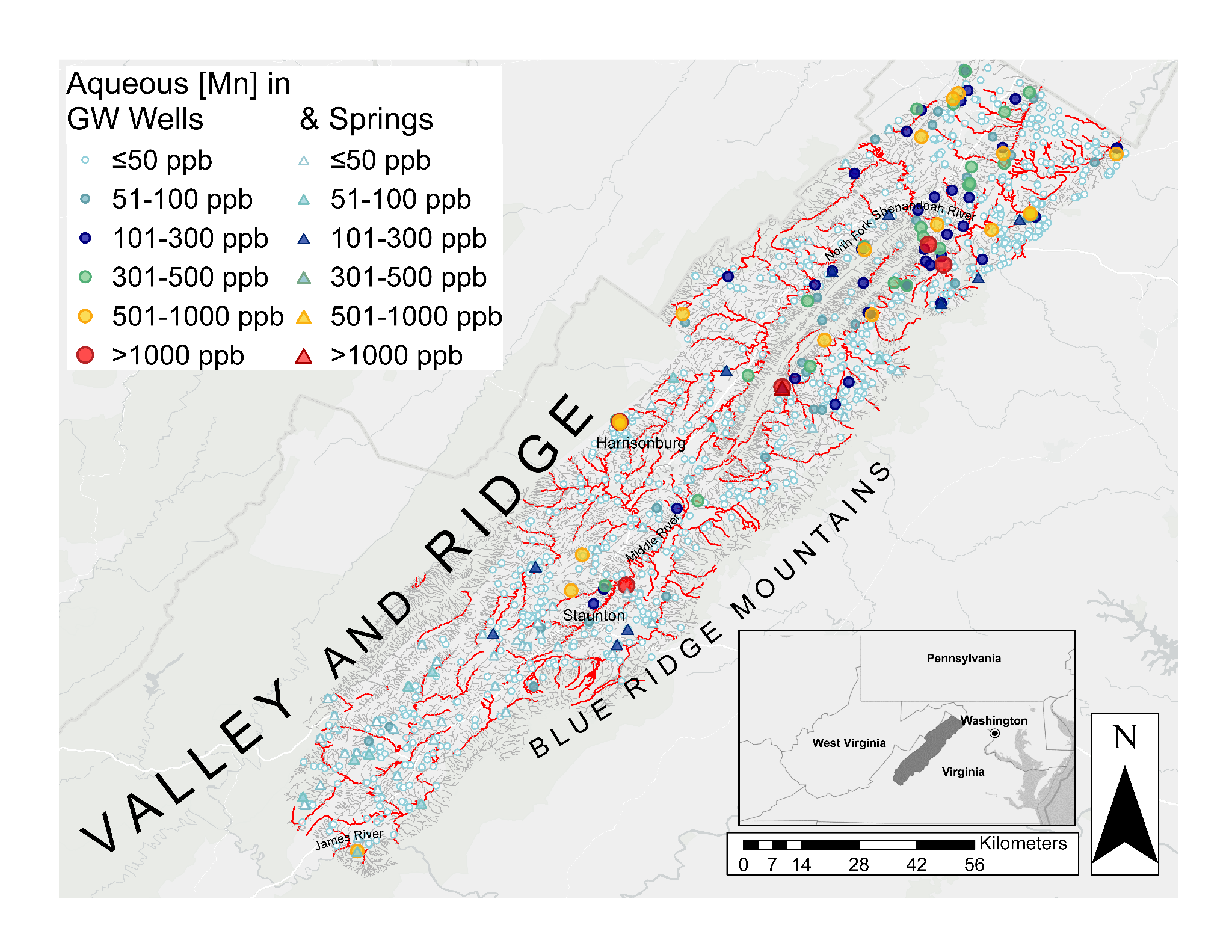


**Figure S7.** Spatial distribution of sampling sites for springs (triangles) and water wells (circles) from private drinking water sources from the VAHWQP data set and the USGS groundwater well network (circles) overlying surface waters (gray lines or, if assessed as impaired, red lines). Sites are color coded by [Mn]_aq_, with values below the threshold of concern for chronic aqueous Mn exposure of 100 ppb as blue, those between 101-300 ppb (yellow), 301-500 ppb (orange), 501-1000 ppb (red), and >1000 ppb (dark red).

**
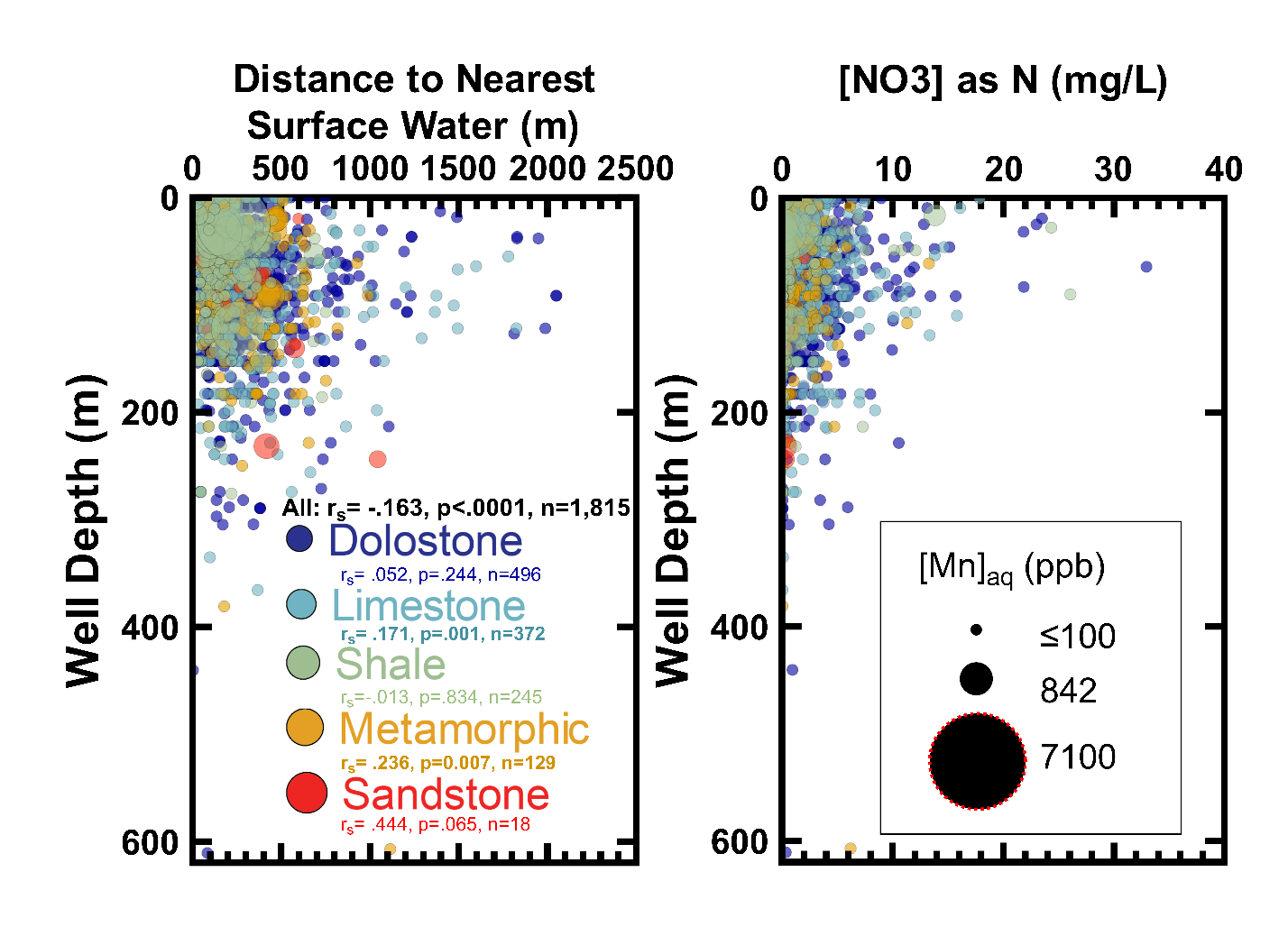
Figure S8.** Well Depth as a function of distance to the nearest surface water body and of nitrate concentrations, with wells in aquifers with primary lithologies comprised of dolostone (dark blue), limestone (bright blue), shale (green), metamorphic (yellow), and sandstone (red). Symbol size varies as a function of aqueous [Mn], with the smallest symbols less than or equal to 100 ppb aqueous Mn. Spearman correlation coefficients (r_s_), total number (n), and two-tailed p values reported for the data set as a whole (denoted as All: r_s_) and for each aquifer primary rock subgroup, with statistically significant coefficients bolded.


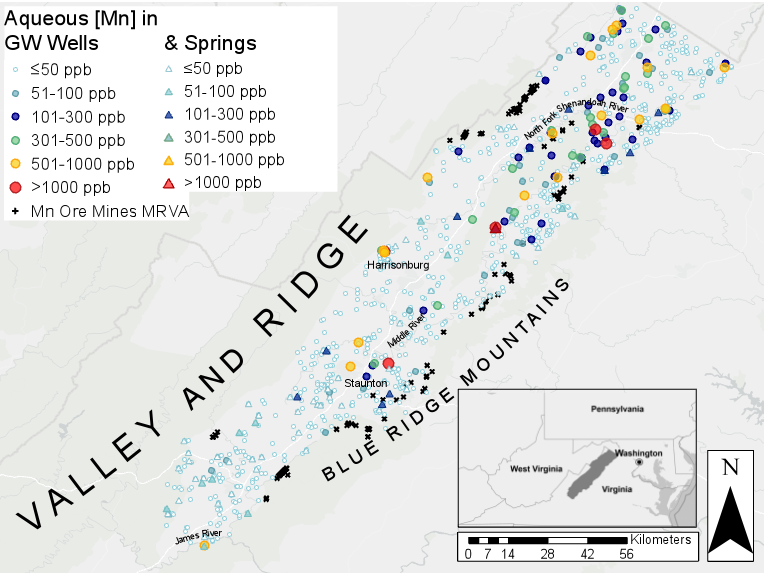


**Figure S9.** Spatial distribution of sampling sites for springs (triangles) and water wells (circles) from private drinking water sources from the VAHWQP data set and the USGS groundwater well network (circles) overlying the presence of Mn ore mines. Sites are color coded by [Mn]_aq_, with values below the threshold of concern for chronic aqueous Mn exposure of 100 ppb as blue, those between 101-300 ppb (yellow), 301-500 ppb (orange), 501-1000 ppb (red), and >1000 ppb (dark red).

**
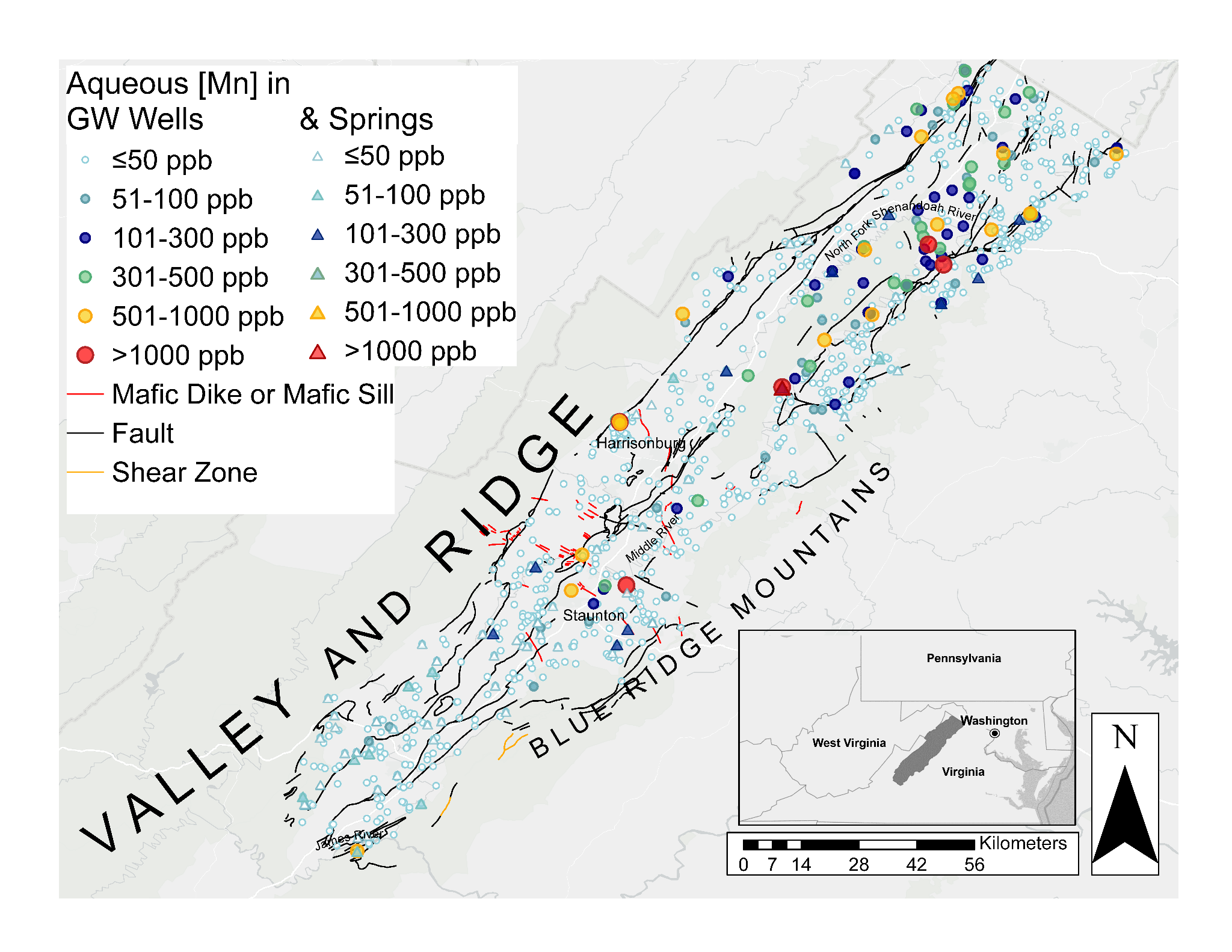
**

**Figure S10.** Spatial distribution of sampling sites for springs (triangles) and water wells (circles) from private drinking water sources from the VAHWQP data set and the USGS groundwater well network (circles) overlying the presence of mafic dikes and sills (red lines), faults (black lines), and shear zones (orange lines). Sites are color coded by [Mn]_aq_, with values below the threshold of concern for chronic aqueous Mn exposure of 100 ppb as blue, those between 101-300 ppb (yellow), 301-500 ppb (orange), 501-1000 ppb (red), and >1000 ppb (dark red).


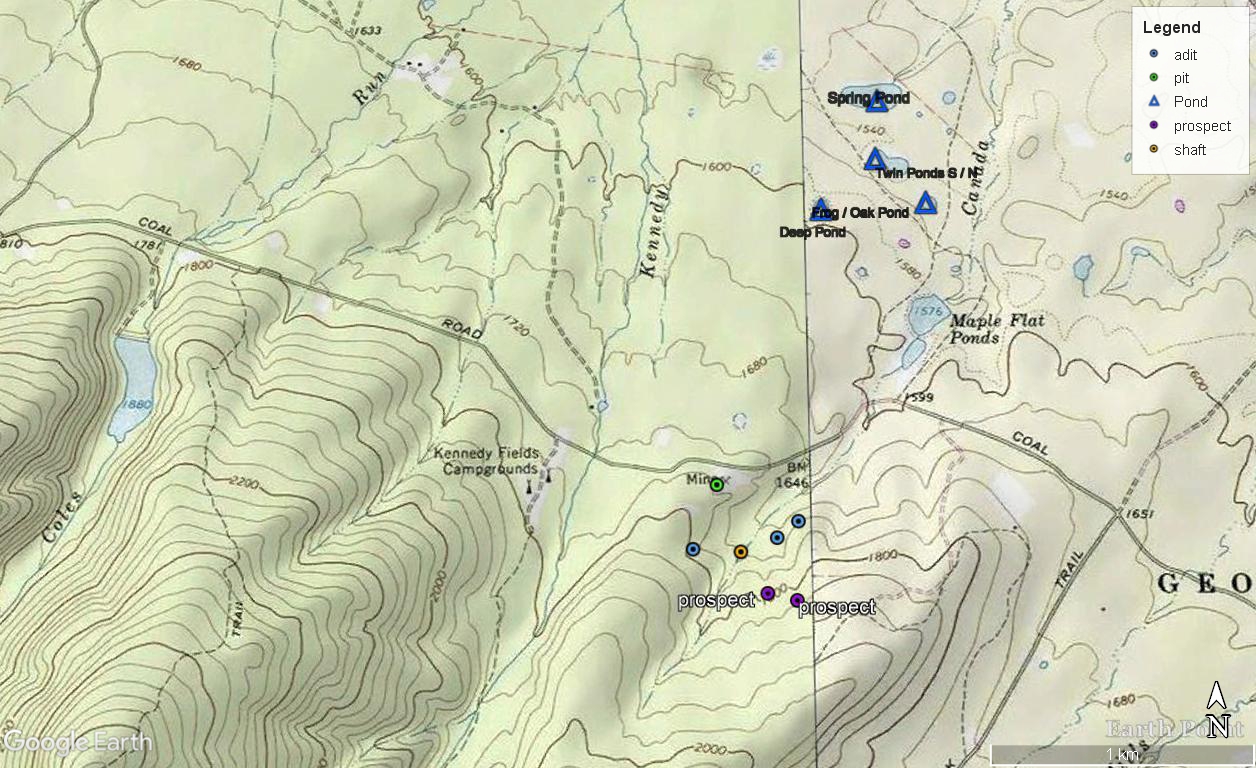


**Figure S11.** Map of Maple Flats Ponds Complex sites that were sampled for water geochemistry and soils, and nearby historic Kennedy Mn ore mine locations.

**
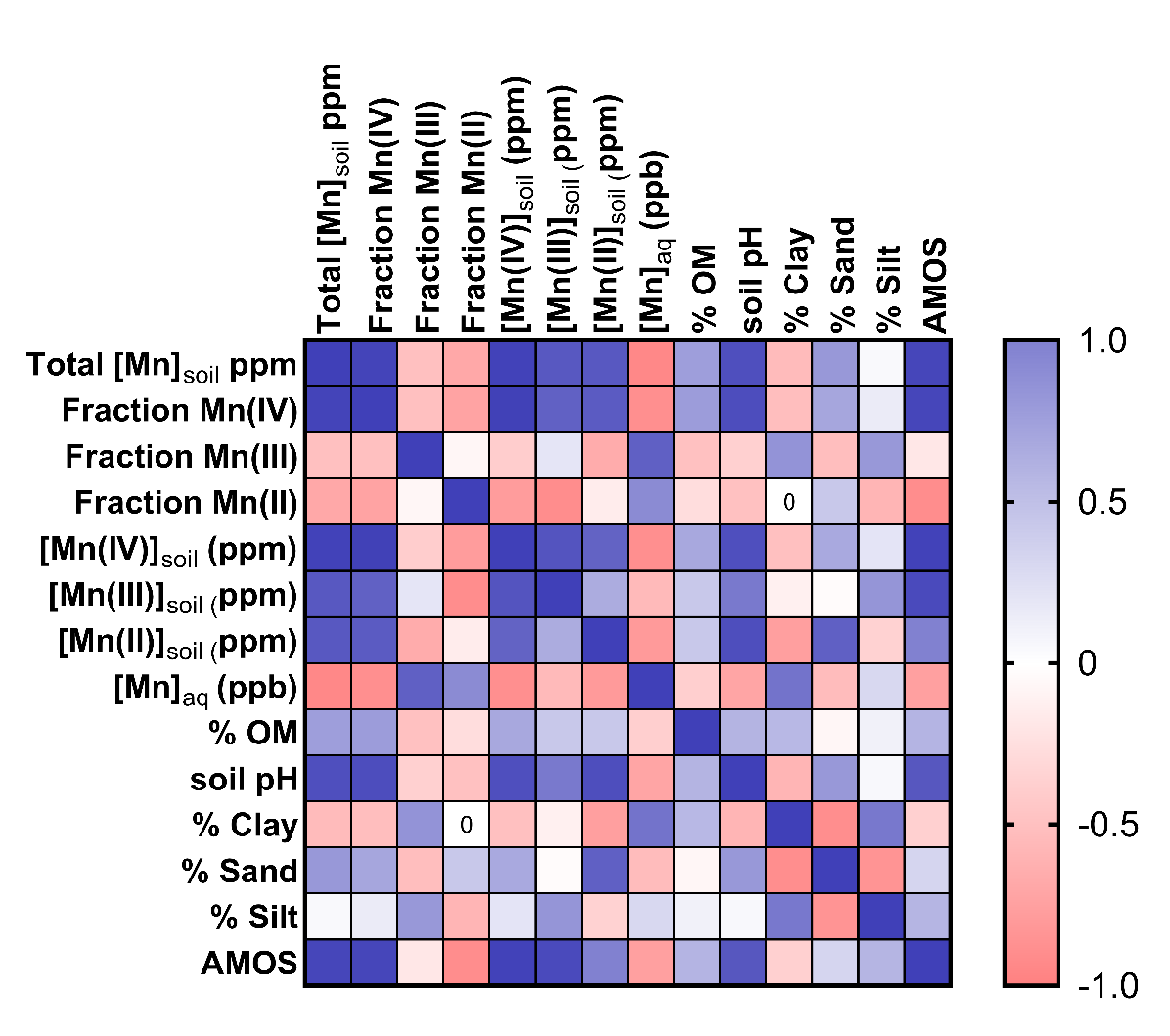
Figure S12.** Spearman’s Rho correlation matrix for the 9 soil samples analyzed by Mn K-edge X-ray absorption near edge structure (XANES) spectroscopy and the suite of soil parameters. “% OM” refers to percent organic matter and “AMOS” refers to average Mn oxidation state as calculated by XANES spectra linear combination fits. Parameters that are not correlated are white, while those that are positively correlated are blue and those negatively correlated appear as red.

**Table S1.** Nonparametric test statistics for aqueous Mn in primary aquifer rock types in the Shenandoah Valley for water wells and springs.

|  |  |  |  |  |  |  |  |  |
| --- | --- | --- | --- | --- | --- | --- | --- | --- |
| **Aquifer Primary Rock Type** | | **Kruskal-Wallis**  **p-values^a^:** | **Wilcoxon test p-values^b^:** | **Dolostone** | **Limestone** | **Metamorphic** | **Sandstone** | **Shale** |
| ***Water Wells*** | | <2 x 10^-16^ |  |  |  |  |  |  |
| Dolostone | |  |  | - | - | - | - | - |
| Limestone | |  |  | **<0.001^c^** | - | - | - | - |
| Metamorphic | |  |  | 0.4925 | **<0.001** | - | - | - |
| Sandstone | |  |  | **<0.001** | **<0.001** | 0.1062 | - | - |
| Shale | |  |  | **<0.001** | **<0.001** | **0.0049** | 0.1062 | - |
|  | |  |  |  |  |  |  |  |
| ***Springs*** | | 0.00607 |  |  |  |  |  |  |
| Dolostone | |  |  | - | - | - | ^d^ | - |
| Limestone | |  |  | 0.3283 | - | - |  | - |
| Metamorphic | |  |  | **<0.001** | **0.0135** | - |  | - |
| Shale | |  |  | **0.0243** | 0.1198 | 0.3767 |  | - |

^a^Kruskal-Wallis tests (α = 0.05) performed for wells and springs, thus finding aquifer lithology in both wells and springs are statistically significantly different.

^b^Wilcoxon tests (α = 0.05) performed for each aquifer pairing.

^c^Statistically significant Wilcoxon tests are bolded.

^d^No values are reported for sandstone springs, as no sandstone springs were in the data sets used for this study as described in Section 3.1).

**Table S2.** Descriptive statistics of aqueous Fe concentrations in the Shenandoah Valley for water wells and springs by most likely aquifer primary rock types.

| *Percentiles for aqueous [Fe] (ppb)* | | | | | | | | |  |
| --- | --- | --- | --- | --- | --- | --- | --- | --- | --- |
| **Aquifer Primary Rock Type** | **#** | **Maximum** | **10^th^** | **25^th^** | **50^th^**  **(Median)** | **75^th^** | **90^th^** | **Mean** | **% > 100 ppb Fe** |
| ***Water Wells*** |  |  |  |  |  |  |  |  |  |
| Dolostone | 687 | 9300 | BDL^a^ | BDL | 2.9 | 15.2 | 42.1 | 80 ± 60 | 5.8% |
| Limestone | 541 | 5240 | BDL | BDL | BDL | 13.4 | 40.4 | 53 ± 350 | 3.5% |
| Metamorphic | 193 | 3838 | BDL | BDL | 10.5 | 61.0 | 393.1 | 160 ± 530 | 18.6% |
| Sandstone | 41 | 2795 | BDL | 3.2 | 26.2 | 250.0 | 2194 | 420 ± 820 | 31.7% |
| Shale | 353 | 18620 | BDL | 2.0 | 17.7 | 85.2 | 549.0 | 330 ± 1400 | 23.5% |
|  |  |  |  |  |  |  |  |  |  |
| ***Springs*** |  |  |  |  |  |  |  |  |  |
| Dolostone | 37 | 137 | BDL | BDL | 3.7 | 21.6 | 57.2 | 20 ± 30 | 5.4% |
| Limestone | 41 | 1011 | BDL | BDL | 2.9 | 39.5 | 130.0 | 50 + 170 | 9.8% |
| Metamorphic | 9 | 2070 | 20.6 | 22.8 | 78.5 | 610.0 | 2070 | 400 ± 700 | 44.4% |
| Shale | 32 | 2844 | BDL | 2.4 | 31.4 | 3563 | 14360 | 3000 ± 6000 | 30.9% |

^a^BDL for ‘below detection limit’ of 1 ppb Mn for samples analyzed by ICP-MS.

**Table S3.** Average aqueous Mn concentrations and associated parameters by soil map units.

| **Soil Map Units** | **Hagerstown-**  **Duffield-**  **Clarksburg** | **Myersville-**  **Catoctin** | **Frederick-Carbo** | **Lowell-**  **Frederick-**  **Chilhowie-**  **Carbo** | **Shottower-**  **Laidig** | **Moomaw-**  **Jefferson-**  **Alonzville** | **Hayesville** | **Wallen-**  **Lily-**  **Drypond-**  **Dekalb** | **Litz-**  **Groseclose** | **Weikert-**  **Berks** |
| --- | --- | --- | --- | --- | --- | --- | --- | --- | --- | --- |
| Soil Order | Alfisol | Alfisol-  Inceptisol Mix | Alfisol-  Ultisol Mix | Alfisol-  Ultisol Mix | Ultisol | Ultisol | Ultisol | Inceptisol-  Ultisol Mix | Inceptisol-  Ultisol Mix | Inceptisol |
| ***Wells***  Number | 286 | 94 | 555 | 191 | 92 | 222 | 13 | 87 | 2 | 273 |
| Mean (ppb) | 8 | 21 | 32 | 4 | 5 | 29 | 42 | 100 | 0.4 | 109 |
| Std Dev (ppb) | 50 | 70 | 330 | 20 | 20 | 120 | 140 | 220 | 0.5 | 340 |
| Max  (ppb) | 555.4 | 430.6 | 7100 | 340.0 | 124.8 | 1348 | 517.0 | 1102 | 0.8 | 4125 |
| ***Springs***  Number | 5 | 6 | 42 | 13 | 5 | 13 | 1 | 6 | - | 6 |
| Mean (ppb) | 0.6 | 19 | 2 | 0.7 | 2 | 6 | 6 | 228 | - | 4 |
| Std Dev (ppb) | 0.4 | 25 | 5 | 1.3 | 3 | 9 | - | 549 | - | 10 |
| Max  (ppb) | 1.090 | 59.32 | 24.00 | 4.563 | 6.444 | 26.96 | 5.612 | 1348 | - | 24.11 |
